# Supplementary material for: Aggregation controlled by condensate rheology
Source: Biophys J. 2022 Nov 11;122(1):197–214. doi: 10.1016/j.bpj.2022.11.009 (PMC9822804; doi:10.1016/j.bpj.2022.11.009)
Supplement: Document S2. Article plus supporting material [file mmc2.pdf]

Biophysical Journal
Article

Aggregation controlled by condensate rheology

Wolfram Pönisch,[1] Thomas C. T. Michaels,[2,3,4,*] and Christoph A. Weber[5,6,7,*]
[1]Department of Physiology, Development and Neuroscience, University of Cambridge, Cambridge, United Kingdom; [2]Laboratory for Molecular Cell Biology, University College London, London, United Kingdom; [3]Medical Research Council Laboratory for Molecular Cell Biology, University College London, London, United Kingdom; [4]Department of Biology, Institute of Biochemistry, ETH Zurich, Zurich, Switzerland; [5]Max Planck Institute for the Physics of Complex Systems, Dresden, Germany; [6]Center for Systems Biology Dresden, Dresden, Germany; and [7]Faculty of Mathematics, Natural Sciences, and Materials Engineering, Institute of Physics, University of Augsburg, Augsburg, Germany

ABSTRACT Biomolecular condensates in living cells can exhibit a complex rheology, including viscoelastic and glassy behavior. This rheological behavior of condensates was suggested to regulate polymerization of cytoskeletal filaments and aggregation of amyloid fibrils. Here, we theoretically investigate how the rheological properties of condensates can control the formation of linear aggregates. To this end, we propose a kinetic theory for linear aggregation in coexisting phases, which accounts for the aggregate size distribution and the exchange of aggregates between inside and outside of condensates. The rheology of condensates is accounted in our model via aggregate mobilities that depend on aggregate size. We show that condensate rheology determines whether aggregates of all sizes or dominantly small aggregates are exchanged between condensate inside and outside on the timescale of aggregation. As a result, the ratio of aggregate numbers inside to outside of condensates differs significantly. Strikingly, we also find that weak variations in the rheological properties of condensates can lead to a switch-like change of the number of aggregates. These results suggest a possible physical mechanism for how living cells could control linear aggregation in a switch-like fashion through variations in condensate rheology.

SIGNIFICANCE The intracellular space can be organized through phase-separated condensates that often exhibit rheological properties reminiscent of complex fluids. These condensates can affect biochemical processes, such as the formation of linear aggregates, in particular biofilaments or amyloids. Here, we propose a theoretical model for how condensate rheology can control the irreversible formation of linear aggregates. A key finding is that size and number of aggregates change in a switch-like fashion upon weak changes in condensate rheology. Our model paves the way to unravel the physiochemical mechanisms of how the rheology of condensates can control aberrant protein aggregation. Such mechanisms may explain how rheological changes, such as aging or the transition to dormancy, give rise to diseases related to protein aggregation.

INTRODUCTION

The formation of linear aggregates plays an important role in many biological processes. Examples are biofilm formation (1,2), the assembly of cytoskeletal filaments (3–5), and amyloids (6). The latter process is involved in a wide range of common and currently incurable diseases, such as Alzheimer's, Parkinson's, and amyloidosis (7–9).

Various theoretical models were proposed that capture key steps of the aggregation kinetics in vitro. An example is the pioneering model of Oosawa and Asakura for polymerization. In this model, linear aggregates can form via primary nucleation and grow at their ends. This model was successfully applied to actin and tubulin polymerization (3,10). Ferrone and Eaton extended this model by introducing secondary pathways and applied to sickle hemoglobin polymerization (11,12). More recently, related models were applied to describe amyloid formation (13–17). All such models capture the kinetics of aggregation in homogeneous environments.

However, many of such aggregation processes occur in living cells and living cells are strongly heterogeneous environments. The heterogeneity of intracellular space is for example due to condensates that form by phase separation (18–20). Such condensates can for example emerge as a response to cellular stress (21–23) and play a vital role during many biochemical processes. Examples are enrichment

Submitted November 5, 2021, and accepted for publication November 4, 2022.

*Correspondence: thomas.michaels@bc.biol.ethz.ch or christoph.weber@physik.uni-augsburg.de

Editor: Dimitrios Vavylonis.
https://doi.org/10.1016/j.bpj.2022.11.009

© 2022 Biophysical Society.
This is an open access article under the CC BY license (http://creativecommons.org/licenses/by/4.0/).

of proteins (24,25), acceleration of gene expression (26), and enhanced drug resistance (27). Recent experimental evidence suggests that condensates also influence the formation of aggregates. For example, phase-separated compartments affect the self-assembly of DNA nanotubes (28) and can initiate or inhibit the formation of cytoskeletal filaments (24,29–31) and amyloid fibrils (32).

Recently, a theoretical model was proposed for the irreversible aggregation kinetics inside and outside of a condensate (33). This model restricts to the case where exclusively monomers are exchanged between the condensate and its environment, but neglects aggregate diffusion. Although aggregates diffuse more slowly than small monomers in liquid phases due to their larger viscous drag, aggregates still get exchanged through the phase boundary of the condensate. However, many protein condensates exhibit complex rheological properties, such as viscoelastic behavior or even glass-like aging (34,35). As a result of such complex rheology, the exchange of big aggregates can be significantly suppressed on the timescale of aggregation and thereby alter the aggregation kinetics. It remained unclear to which degree the aggregation kinetics is affected by condensate rheology and what happens if the rheological properties change.

In our study, we investigate how the exchange of both monomers and aggregates between the two coexisting phases affects the kinetics of linear aggregation and how this exchange depends on condensate rheology. The rheological properties of condensates are accounted for by an aggregate mobility ξ_i that depends on aggregate size i (see Eq. 12). Using our model, we find two distinct regimes with qualitatively different behaviors for the size distribution of linear aggregates. The regimes depend on whether all aggregates or only small aggregates are exchanged faster than the aggregation timescale. Another key factor is the partitioning between the phases, which is determined whether aggregates either interact over their complete length with the phase-separating material or exclusively with their ends. In our work, we develop the corresponding thermodynamic and kinetic theory to study how condensate rheology can affect irreversible formation of linear aggregates that interact differently with the phase-separated material. We report differences in size distributions and aggregate mass ratio between the two phases and reveal a switch-like change of the aggregation kinetic upon weak changes in condensate rheology.

METHODS

In the following, we introduce a set of master equations to describe linear aggregation in the presence of a phase-separated condensate with various rheological properties. We also derive the governing relationships for how monomers and aggregates partition and are transported between the inside and outside of the condensate. The rate of aggregate exchange between the phases is considered to have a specific dependence on aggregate size, which is a result of a specific condensate rheology. Thus, the resulting model allows us to discuss how rheological properties of condensates can affect the kinetics of aggregation.

Master equation of irreversible linear aggregation

To describe the temporal evolution of irreversible linear aggregation, we introduce a set of master equations for the aggregate concentration $c_i^{(\alpha)}$ (units of molar concentration M). Here, i indicates the aggregate size, while α denotes the inside ($\alpha = \mathrm{I}$) and outside ($\alpha = \mathrm{II}$) of the condensate, also called phase I and phase II. The evolution of monomer and aggregate concentrations in each phase is given by

$$\frac{dc_1^{(\alpha)}}{dt} = -k_1^{(\alpha)}\left(c_1^{(\alpha)}\right)^{n_1} n_1 - 2k_+^{(\alpha)} c_1^{(\alpha)} \sum_{i=n_1}^{\infty} c_i^{(\alpha)} - k_2^{(\alpha)}\left(c_1^{(\alpha)}\right)^{n_2} n_2 \sum_{i=n_2}^{\infty} i\, c_i^{(\alpha)} + \frac{J_1^{(\alpha)}}{V^{(\alpha)}} \tag{1a}$$

$$\frac{dc_i^{(\alpha)}}{dt} = k_1^{(\alpha)}\left(c_1^{(\alpha)}\right)^{n_1}\delta_{i,n_1} + 2k_+^{(\alpha)} c_1^{(\alpha)} c_{i-1}^{(\alpha)}\delta_{i>n_1} - 2k_+^{(\alpha)} c_1^{(\alpha)} c_i^{(\alpha)}\delta_{i\geq n_1} + k_2^{(\alpha)}\left(c_1^{(\alpha)}\right)^{n_2}\delta_{i,n_2}\sum_{j=n_2}^{\infty} j\, c_j^{(\alpha)} + \frac{J_i^{(\alpha)}}{V^{(\alpha)}},\ i>1. \tag{1b}$$

The terms, including the rate constant $k_1^{(\alpha)}$, correspond to primary nucleation, leading to the initial formation of aggregates. During primary nucleation, n_1 monomers assemble one aggregate of size n_1, where n_1 is called the reaction order. Monomers can also bind to the ends of aggregates with a rate constant $k_+^{(\alpha)}$, creating aggregates with a size $i > n_1$. In addition, the subunits of aggregates of size $i \leq n_2$ act as nucleation sites for new aggregates of size n_2 to form by a process called secondary nucleation occurring with a rate constant $k_2^{(\alpha)}$. In general, the rate constants $k_1^{(\alpha)}$, $k_2^{(\alpha)}$, and $k_+^{(\alpha)}$ can be different between both phases I and II. For example, if reactions between monomers and aggregates are limited by their diffusion, the rates might depend on the rheology of the two phases. For simplicity, we consider phase-independent rate constants and write k_1, k_2, and k_+. In Fig. 1 *A*, we provide a graphical representation of primary and secondary nucleation and aggregate elongation.

The master equations Eqs. 1a and 1b are motivated by previous studies of cytoskeletal polymerization (3,10) and amyloid fibril aggregation (14,15,17,36). Equations 1a and

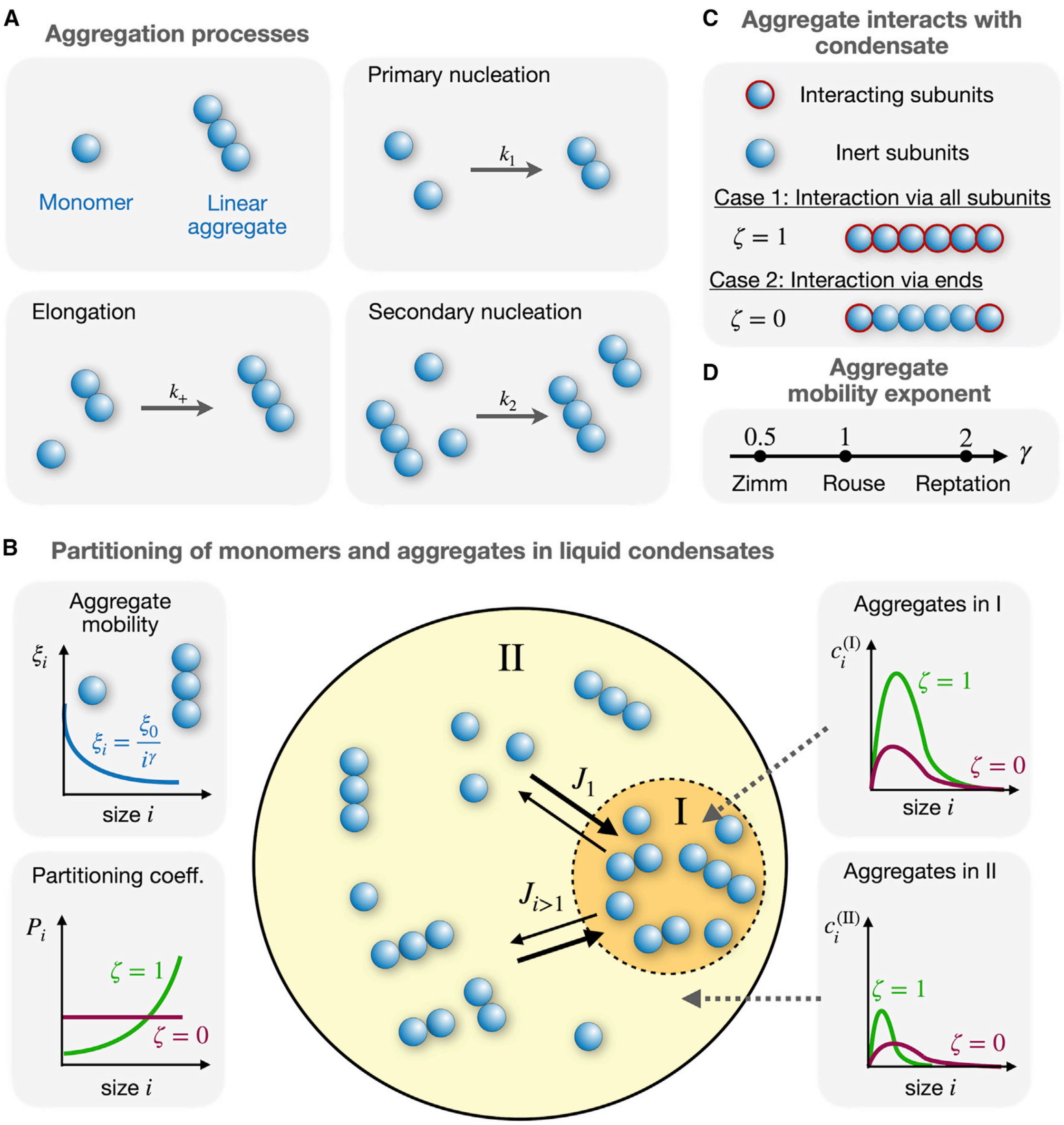

FIGURE 1 Illustration of our model for linear aggregation in the presence of a phase-separated condensates with varying rheology. (*A*) Elementary processes driving the formation of monomers to linear aggregates: primary nucleation with rate constant k_1, elongation with rate constant k_+, and secondary nucleation with rate constant k_2. (*B*) Monomers and linear aggregates within phase-separated condensates. Monomers and aggregates of size i are exchanged between phase (I) to phase (II) by diffusion with a rate J_i (units: 1/s). The aggregate size dependence of the aggregate mobility ξ_i (Eq. 12) and the partition coefficient P_i (Eq. 4) control the aggregation kinetics and thus the profile of the aggregate concentrations $c_i^{(\alpha)}$ inside and outside of the condensate, $\alpha = $ (I), (II). Both also depend on how linear aggregates interact with the phase-separating components A and B. (*C*) Either all aggregate subunits interact with the condensate (binding parameter $\zeta = 1$) or only aggregate ends interact with the condensates ($\zeta = 0$). (*D*) Sketch of the different regimes of the mobility exponent γ of the linear aggregates, dependent on the aggregate concentration and hydrodynamic interactions. To see this figure in color, go online.

1b govern the temporal dynamics of monomers and Eq. 1b describes the temporal dynamics of the aggregates of length $i > 1$. Phase coexistence dictates the use of the chemical activities $a_i^{(\alpha)} = \gamma_i^{(\alpha)} c_i^{(\alpha)}$ of the aggregates in the reaction steps, where $\gamma_i^{(\alpha)}$ denotes the chemical activity coefficient. This accounts for the coupling between aggregate partitioning and nucleation and growth of aggregates (37). However, in Appendix D, we show that both processes can decouple for irreversible processes leading to Eqs. 1a and 1b.

There is a diffusive total exchange rate J_i that describes the exchange of aggregates of size i between the interior and exterior of the condensate (see Fig. 1 *B*). The total exchange rate conserves monomer and aggregate mass as well as particle numbers, and thus obeys $J_i^{(I)} = -J_i^{(II)} \equiv -J_i$. Here, we consider the case of dilute aggregates which implies that the exchange of aggregates between the inside and outside does not affect the condensate volumes $V^{(I)} = V - V^{(II)}$, where V is the system volume.

The stationary state of Eqs. 1a and 1b is a nonequilibrium steady state. In our work, we explicitly focus on aggregation kinetics where the timescale of reaching thermodynamic equilibrium exceeds the timescale of interest of most monomers being depleted. A well-studied example for the latter case is the aggregation of amyloid fibrils. Motivated by this class of aggregation processes, we consider a special case where the reaction kinetics for nucleation and growth in each phase (i.e., $dc_i^{(\alpha)}/dt - J_i^{(\alpha)}/V^{(\alpha)}$) have the same mathematical form as in a homogeneous system and are independent of partitioning of monomers and aggregates.

We also introduce the aggregate number concentration, defined by

$$c_A^{(\alpha)} = \sum_{i=n_1}^{\infty} c_i^{(\alpha)} \tag{2}$$

and the aggregate mass concentration

$$M_A^{(\alpha)} = \sum_{i=n_1}^{\infty} i\, c_i^{(\alpha)}, \tag{3}$$

which correspond to the zeroth and first moment of the aggregate concentration $c_i^{(\alpha)}$, respectively. Details of the numerical solution of the master equations are given in Appendix A.

The interplay of linear aggregation and the flux of aggregates between the inside and outside of the condensate controls the aggregate concentration profile within the two phases I and II. This interplay depends on the partitioning of monomers and aggregates as well as on the rate of diffusive exchange. In the following, we derive an expression for the partition coefficient of aggregates as a function of their size, and then derive the flux law for monomer and aggregate exchange.

Phase separation and partitioning of linear aggregates

We define the partition coefficient at phase equilibrium as

$$P_i = \frac{c_i^{(\mathrm{I,eq})}}{c_i^{(\mathrm{II,eq})}}, \tag{4}$$

of aggregates of size i and concentrations $c_i^{(\alpha,\mathrm{eq})}$ are the equilibrium concentrations inside ($\alpha = \mathrm{I}$) or outside ($\alpha = \mathrm{II}$) of the condensate. At phase equilibrium, chemical potentials, $\mu_i \equiv i\nu\partial f/\partial\varphi_i$ (ν is the molecular volume of a monomer), are equal between both phases:

$$\mu_i^{(\mathrm{I})} = \mu_i^{(\mathrm{II})}. \tag{5}$$

To determine the chemical potential, we consider an incompressible mixture composed of two phase-separating components A and B, and aggregating monomers. For example, phase I is A-rich (B-poor), while phase II is B-rich (A-poor). The monomers are prone to undergo irreversible aggregation into linear aggregates of size i. We study the case where the monomers and the resulting aggregates are highly diluted with respect to components A and B, which is consistent with physiological conditions, e.g., for amyloid-β monomers and fibrils (see Appendix B). To derive the partitioning of the aggregates, we use a free energy that is qualitatively similar to the Flory-Huggins free energy (38,39), but does not rely on mean field approximations of the highly dilute linear aggregates (40). The derivation of the free energy of dilute aggregates of size i is provided in Appendix C. This free energy density is given by

$$\begin{aligned} f = \frac{k_B T}{\nu}\Bigg[&\frac{\varphi}{n_A}\ln\varphi + \frac{\left(1-\varphi-\sum_{j=1}^{M}\varphi_j\right)}{n_B} \\ &\times \ln\left(1-\varphi-\sum_{j=1}^{M}\varphi_j\right) + \sum_{i=1}^{M}\left(\frac{\varphi_i}{i}\ln\left(\frac{\varphi_i}{i}\right) - \frac{\varphi_i}{i}\right. \\ &\left. - \frac{\varphi_i}{i}\ln\Omega_i^{(\mathrm{conf})}\right) + \chi\varphi\left(1-\varphi-\sum_{j=1}^{M}\varphi_j\right) \\ &+ \sum_{i=1}^{M}\left(\chi_{i,A} i^{\zeta-1}\varphi_i\varphi + \chi_{i,B} i^{\zeta-1}\varphi_i(1-\varphi)\right) \\ &+ \sum_{i=1}^{M}\Delta w\varphi_i\frac{i-1}{i}\Bigg], \end{aligned} \tag{6}$$

where $\varphi_A = \varphi$ and $\varphi_B = 1 - \varphi - \sum_{j=1}^{M}\varphi_j$ denote the volume fractions of components A and B, respectively, and n_A and n_B are the sizes of components A and B measured in terms of the molecular volume of monomers denoted as ν. Moreover, φ_i is the volume fraction of the aggregates of size i and M is the maximal aggregates size, i.e., $1 \le i \le M$. Note that $\varphi_i \ll 1$ since the linear aggregates are dilute.

The first two terms in Eq. 6 correspond to the mixing entropy of the components A and B. The sum over the aggregate size i involves contributions from the mixing entropy of the aggregates and entropic contributions related to the number of possible aggregate configurations, $\Omega_i^{(\mathrm{conf})}$, which depends on the size i. The terms, including the parameters χ, $\chi_{i,A}$, and $\chi_{i,B}$, represent enthalpic contributions due to the interactions between the components A, B and the aggregates of size i with the respective interaction parameters χ, $\chi_{i,A}$, and $\chi_{i,B}$. Since the linear aggregates are considered to be dilute, we have neglected aggregate-aggregate interactions in the free energy. In some experimental studies on the molecular weight dependence of the interaction parameter in polymer-polymer-good-solvent systems it has been suggested that the interaction parameters depend on polymer length by the equations $\chi_{i,A} = \chi_A/\sqrt{i}$ and

$\chi_{i,B} = \chi_B/\sqrt{i}$ (41). For simplicity, we will neglect any aggregate size dependence of the interaction parameters, setting $\chi_{i,A} = \chi_A$ and $\chi_{i,B} = \chi_B$, as suggested in experimental measurements in polymer-liquid crystal mixtures (42), but we note that our model can be extended in a straightforward way to account for different size dependencies of $\chi_{i,A}$ and $\chi_{i,B}$.

We also introduce the parameter ζ, which we refer to as binding parameter in the following. The binding parameter ζ characterizes how the aggregate subunits interact with the phase-separating components A and B: $\zeta = 1$ corresponds to the case where all subunits can bind to A and B molecules, while $\zeta = 0$ is the case where only the subunits at the linear aggregate ends can bind to A and B molecules (see Fig. 1 *C*). This could play a role for subunits with hydrophobic binding sites that are buried when the subunit is within the bulk of a linear aggregate, but freely available for binding to A and B for subunits at the aggregate ends. The internal free energy Δw describes the free energy of each bond in a polymer of length i. Since both coexisting phases are liquids, Δw is phase independent.

The chemical potential of aggregates of size i can be calculated using Eq. 6, leading to

$$\begin{aligned}\mu_i = k_BT\Bigg[&-\frac{i\ln\left(1-\varphi-\sum_{j=1}^{M}\varphi_j\right)}{n_B}-\frac{i}{n_B}\\&-\ln\Omega_i^{(\mathrm{conf})}+\ln\left(\frac{\varphi_i}{i}\right)+i^{\zeta}\varphi\chi_A+i^{\zeta}(1-\varphi)\chi_B\\&-i\chi\varphi+\Delta w(i-1)\Bigg]\approx k_BT\Bigg[-\frac{i\ln(1-\varphi)}{n_B}-\frac{i}{n_B}\\&-\ln\Omega_i^{(\mathrm{conf})}+\ln\left(\frac{\varphi_i}{i}\right)+i^{\zeta}\varphi\chi_A+i^{\zeta}(1-\varphi)\chi_B\\&-i\chi\varphi+\Delta w(i-1)\Bigg]\end{aligned} \tag{7}$$

At equilibrium, the difference of chemical potentials between two separated phases, (I) and (II) (see Fig. 1 *B*)

$$\begin{aligned}\mu_i^{(\mathrm{I})}-\mu_i^{(\mathrm{II})} = k_BT\Bigg[&\ln\left(\frac{\varphi_i^{(\mathrm{I})}}{\varphi_i^{(\mathrm{II})}}\right)-i^{\zeta}\left(\varphi^{(\mathrm{I})}-\varphi^{(\mathrm{II})}\right)(\chi_B\\&-\chi_A)-i\chi\left(\varphi^{(\mathrm{I})}-\varphi^{(\mathrm{II})}\right)\\&+\frac{i}{n_B}\ln\left(\frac{1-\varphi^{(\mathrm{II})}}{1-\varphi^{(\mathrm{I})}}\right)\Bigg],\end{aligned} \tag{8}$$

is zero. Using this condition, we find for the partition coefficient

$$\begin{aligned}P_i = \exp\Bigg[&i^{\zeta}\left(\varphi^{(\mathrm{I})}-\varphi^{(\mathrm{II})}\right)(\chi_B-\chi_A)+i\chi\left(\varphi^{(\mathrm{I})}-\varphi^{(\mathrm{II})}\right)\\&-\frac{i}{n_B}\ln\left(\frac{1-\varphi^{(\mathrm{II})}}{1-\varphi^{(\mathrm{I})}}\right)\Bigg].\end{aligned} \tag{9}$$

At phase equilibrium, it fulfills

$$P_i = \exp\left[i^{\zeta}\left(\varphi^{(\mathrm{I})}-\varphi^{(\mathrm{II})}\right)(\chi_B-\chi_A)\right]. \tag{10}$$

Since we assume that the aggregate concentration $c_i^{(\alpha)}$ is proportional to the aggregate volume fraction $\varphi_i^{(\alpha)}$, Eq. 10 implies $\varphi_i^{(\mathrm{I})}/\varphi_i^{(\mathrm{II})} = c_i^{(\mathrm{I})}/c_i^{(\mathrm{II})}$. For the binding parameter $\zeta = 1$, the partition coefficient increases monotonically with aggregate size, assuming that $(\varphi^{(\mathrm{I})}-\varphi^{(\mathrm{II})})(\chi_B-\chi_A)>0$, and hence, P_i increases with the molecular weight of aggregates. This increase is in accordance with previously reported experimental studies on hydrophobic compounds in polymer-water mixtures (43) and dilute polymers between cylindrical pores and an exterior solution (44). While, in both studies, the partition coefficient appears to increase exponentially with molecular weights, this is only true for a small range of molecular weights. We also consider the case $\zeta = 0$, corresponding to the limit in which A and B exclusively bind to the aggregate ends (see Fig. 1 *C*) and as a result, the partition coefficient is independent of aggregate size i.

Flux of linear aggregates between the inside and outside of the condensate

Near equilibrium, the total flux of aggregates of size i is proportional to the difference of the corresponding chemical potentials inside and outside the condensate and can be written as (33):

$$J_i = \xi_i\frac{c_i^{\mathrm{I}}+c_i^{\mathrm{II}}}{2}\left(\mu_i^{(\mathrm{I})}-\mu_i^{(\mathrm{II})}\right), \tag{11}$$

where the dependence on c_i^{II} ensures that the mobility ξ_i is constant in the dilute limit. This mobility characterizes how fast a phase of size $(V^{\mathrm{I}})^{1/3}$ is homogenized suggesting a phenomenological relationship to the diffusion coefficient of the form $D_i \sim \xi_i/(V^{\mathrm{I}})^{1/3}$ if the transport of aggregates inside is the rate limiting step.

To account for the impact of condensate rheology on aggregation kinetics, we introduce a mobility that depends on aggregate size i. In the following, we consider a power law dependence of the form:

$$\xi_i = \frac{\xi_0}{i^{\gamma}}, \tag{12}$$

where ξ_0 is the mobility prefactor and γ denotes the exponent of the power law (see Fig. 1, *B* and *D*). In our work,

we distinguish between three fundamentally different rheological behaviors corresponding to different interactions among the polymeric components (Fig. 1 *D*). For polymers interacting via hydrodynamic interactions, the exponent is $\gamma = 0.5$, which corresponds to the Zimm regime. Without hydrodynamic interactions and solely accounting for single polymer friction with the solvent, $\gamma = 1$, is refereed to the Rouse regime. The case $\gamma = 2$ describes polymers that are entangled by neighboring polymers that can escape via reptation (45,46). Please note that such fundamental scaling exponents in the Zimm, Rouse, and reptation regime apply to a homogeneous system, consisting only of one phase. This situtation does not necessarily apply to biological systems, such as the cytoplasm, which is heterogeneous and composed of many different components and condensed phases (47–49). For such complex environments, we rely on a phenomenological description of how the mobility depends on aggregate size i and thus we also consider noninteger valued rheology exponent γ to account for complex polymer melts or micro phase-separated mixtures.

Based on the mobility ξ_i, we can derive a characteristic timescale for the exchange of aggregates between the condensate inside and outside (see Appendix F):

$$\tau_{\mathrm{f}}(i) = \frac{2V^{(\mathrm{I})}}{\xi_i \ln P_i} \propto i^{\gamma-\zeta}. \tag{13}$$

When the binding parameter ζ is equal to the mobility exponent γ, i.e., $\gamma = \zeta$, the characteristic time of aggregate exchange is independent of aggregate size. For $\gamma > \zeta$, larger aggregates are exchanged slower between the condensate inside and outside than small ones, while the opposite holds for $\gamma < \zeta$.

The characteristic exchange of aggregates $\tau_{\mathrm{f}}(i)$ allows us to enforce phase equilibrium depending on aggregate size i. In particular, we consider two limits: on short timescales $t < \tau_{\mathrm{f}}(i)$, aggregates of size i are not exchanged between the phases. In this case, we set the exchange total exchange rate $J_i = 0$, implying that aggregates nucleate and grow in each phase independently of the other phase. In contrast, on large timescales $t \geq \tau_{\mathrm{f}}(i)$, aggregates are at phase equilibrium and thus, at each time point, the relative concentration of aggregates follow the partition coefficient (see Eqs. 4 and 10).

RESULTS AND DISCUSSION

In the following, we investigate the aggregation dynamics of dilute linear aggregates in a system where two phases coexist and study how we can control the outcome of aggregation by varying condensate rheology via changes of the mobility prefactor ξ_0 and exponent γ. To this aim, we solve the master equations (see Eqs. 1a and 1b) numerically; see Appendix A for details. Our theory can describe the irreversible nucleation and growth kinetics of linear aggregates in the presence of phase-separated condensates. In the following, we choose parameters consistent with experimental measurements on Aβ42 monomers forming amyloid fibrils (details see Table 1 and Appendix A). For droplets of sizes around $1 - 10\ \mu$m, we find that the mobility prefactor ξ_0 in Eq. 11 has values around $\xi_0 \approx 10^3 - 10^4 \mu\mathrm{m}^3/\mathrm{s}$ (see Appendix E). For the initial monomer concentration, we pick values that are comparable with in vitro studies on amyloid kinetics, i.e., 4 μM (32). This concentration is far above estimates for in vivo concentrations which are around 10–200 pM (50,51). The choice of a μM-ranged monomer concentration enables us to scrutinize our results by in vitro experiments, where typical aggregation times are in the order of hours. In addition, we consider condensates that are small compared with the system size, $V^{(\mathrm{I})}/V \simeq 10^{-2} \ll 1$, to highlight that even small volumes can have a significant impact on aggregation.

Condensate rheology controls aggregation kinetics and distribution

To identify how condensate rheology affects monomer and aggregate partitioning, we solve the master equations (see Eqs. 1a and 1b) and quantify the main features of the linear aggregate size distribution as a function of the mobility prefactor ξ_0 and exponent γ (see Eq. 12, Figs. 2 and S1).

Initially, we focus on the case $\zeta = 1$, which corresponds to aggregates binding to the phase-separating components A and B with all their subunits. Setting the binding parameter $\zeta = 1$ in Eq. (10) makes the partition coefficient P_i size dependent. We assume that $P_i > 1$, so that monomers and aggregates are accumulated inside the condensate (phase I). We start our investigation by initializing the system exclusively with monomers at their equilibrium concentration inside and outside the condensate, fulfilling $c_1^{(\mathrm{I})}/c_1^{(\mathrm{II})} = P_1$. We then allow the monomers to form aggregates and both monomers and aggregates are exchanged between the inside and outside of the condensate if their exchange time $\tau_{\mathrm{f}} > t$ (see Eq. 13) with the time. The reduction of the monomer concentration leads to monomer and aggregate fluxes between the two phases to fulfill the partition coefficient. We numerically solve the master equations until 99% of the initial total monomer concentration $c_1^{(\mathrm{tot})}(t = 0)$ in the system have assembled into aggregates of size $j > 1$. This threshold allows us to define the point at which most

TABLE 1 Physicochemcial parameters of Aβ42 fibril aggregation estimated by in vitro experiments

Parameter	Aβ42	Ref.
k_+	$3 \times 10^6\ \mathrm{M}^{-1}\ \mathrm{s}^{-1}$	Meisl et al. (62)
k_1	$3 \times 10^{-4}\ \mathrm{M}^{-1}\ \mathrm{s}^{-1}$	Meisl et al. (62)
k_2	$1 \times 10^4\ \mathrm{M}^{-2}\ \mathrm{s}^{-1}$	Meisl et al. (62)
n_1	2	Weber et al. (33)
n_2	2	Weber et al. (33)

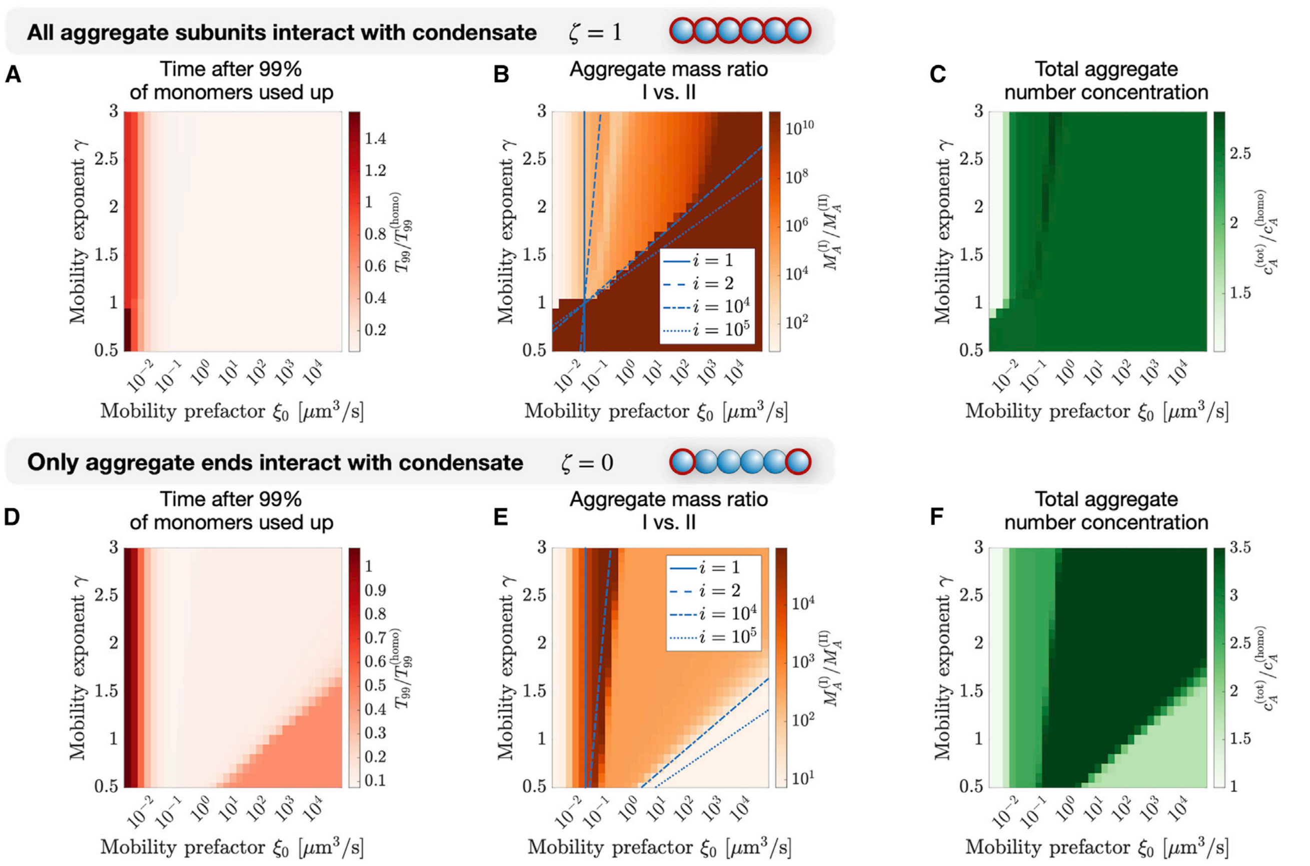

FIGURE 2 Diagrams of how condensate rheology affects linear aggregate distribution. For the binding parameter $\zeta = 1$ (the partition coefficient is given by $P_i = \exp 2\,i$) and $\zeta = 0$ (with $P_i = \exp 2$), the master equations (see Eqs. 1a and 1b) is solved and (*A* and *D*) the aggregation time T_{99}, (*B* and *E*) aggregate mass ratio, and (*C* and *F*) the total aggregate number concentration $c_A^{(\mathrm{tot})}$ are given after the time T_{99}, corresponding to the time it takes 99% of monomers in the system to assemble to linear aggregates. The aggregation time T_{99} is normalized by the time $T_{99}^{(\mathrm{homo})}$ it takes to for the monomers to aggregate in the homogeneous case with only a single phase, but the identical initial total monomer concentration. Analogously, the total aggregate number concentration is normalized by the total aggregate number concentration of the homogeneous case. The quantities are investigated for different values of the mobility exponent γ and prefactor ξ_0 (see Eq. 12). The blue curves in (*B*) show when the aggregation time $T_{99}^{(\mathrm{mono})}$ in the case of exclusive and instantaneous monomer exchange (see supporting material S1.2) and the aggregate exchange time (see Eq. 13) are the same as a function of aggregate sizes *i*. Here, the total initial monomer concentration is $c_1^{(\mathrm{tot})}(t = 0) = 4\ \mu\mathrm{M}$ (see Eq. 14) and the volumes are $V^{(\mathrm{I})} = 10.1\ \mu\mathrm{m}^3$ and $V^{(\mathrm{II})} = 1000\ \mu\mathrm{m}^3$. To see this figure in color, go online.

monomers in the system have assembled into aggregates and the remaining dynamics of the system are dominated by monomer and aggregate exchange between the two phases. The corresponding time T_{99} is determined by the condition $c_1^{(\mathrm{tot})}(t = T_{99}) = 0.01 c_1^{(\mathrm{tot})}(t = 0)$. Here, the total monomer concentration is given by

$$c_1^{(\mathrm{tot})}(t) = \frac{V^{(\mathrm{I})} c_1^{(\mathrm{I})}(t) + V^{(\mathrm{II})} c_1^{(\mathrm{II})}(t)}{V^{(\mathrm{I})} + V^{(\mathrm{II})}}. \tag{14}$$

Since we do not allow for any fragmentation or aggregation processes that are not involving monomers, the aggregation process slows down the fewer monomers are left and stops when no more monomers are available. In Fig. 2 *A*, we show the time T_{99} and we find that this time is dominantly dependent on the mobility prefactor ξ_0 for $\zeta = 1$. For small values of ξ_0, the time approaches the value of the homogeneous system with a single phase only. For small mobility prefactor ξ_0 and small mobility exponent γ, we find that the time can even exceed the aggregation time in the homogeneous case. For higher values of ξ_0, the time T_{99} decreases, implying that condensates accelerate aggregation due to the accumulation of monomers and linear aggregates within phase I, as described previously (33). For $\xi_0 > 0.01\ \mu\mathrm{m}^3/\mathrm{s}$, the time T_{99} is not dependent on the mobility exponent γ. This result suggests that the time T_{99} is dominantly influenced by the monomer exchange, which is independent of γ, and not by the aggregate exchange, which is controlled by the mobility exponent γ.

We next investigated the distribution of aggregate mass in the two phases by looking at the ratio of the aggregate mass concentrations (see Eq. 3 and Fig. 2 *B*) and the aggregate mass concentration outside of the condensate in phase II (see Fig. S1 *A*). We find that independently of the mobility

and, since $P_i > 1$, the mass ratio is higher than 1, hence aggregates are accumulated within the condensate (phase I). In addition, we find two regimes: In the first regime, given by small values of γ and large enough values of ξ_0, the mass ratio is very high and exceeds 10^{10} and the aggregate mass concentration outside of the condensate is around 10^{-8} times smaller than the concentration for the single phase case. This corresponds to essentially all linear aggregates being located within the condensate. For higher values of γ and smaller values of ξ_0, the mass ratio is in the order of $10^2 - 10^4$ and considerably smaller. We observe similarly pronounced differences in the ratio of mean aggregate size between the inside and outside of the condensate (see Fig. S1 *B*). We always observe that linear aggregates within the condensate (in phase I) have a larger mean size than aggregates outside of the condensate (in phase II) but, for small enough γ, the size ratio is considerably higher than for larger γ.

The mass ratio and the mean aggregate size ratio are both quantities that have not yet equilibrated after 99% of monomers have aggregated. The ratio of aggregates outside and inside of the condensate does not necessarily fulfill the partition coefficient P_i (see Eq. 10) yet and the aggregate flux is then nonzero. Thus, we consider the total aggregate number concentration,

$$c_A^{(\mathrm{tot})}(t) = \frac{V^{(\mathrm{I})} c_A^{(\mathrm{I})}(t) + V^{(\mathrm{II})} c_A^{(\mathrm{II})}(t)}{V^{(\mathrm{I})} + V^{(\mathrm{II})}}. \tag{15}$$

This quantity describes the total concentration of aggregates in the total volume, $V^{(\mathrm{I})} + V^{(\mathrm{II})}$, and is near its equilibrium value at $t = T_{99}$ since most monomers have assembled to aggregates and the irreversible aggregate production has almost ended. We show the total aggregate number concentration at time T_{99} in Fig. 2 *C*. We find that, analogously to the time T_{99}, the final total concentration $c_A^{(\mathrm{tot})}(t)$ is only weakly dependent on the mobility exponent γ, but dependent on the mobility prefactor ξ_0. For $\xi_0 < 0.01\ \mu\mathrm{m}^3/\mathrm{s}$, we find that the aggregate number concentration is similar to the homogeneous case, except for $\gamma < 1$. In addition, we find a weak nonmonotonous dependence of the number concentration on γ for $\xi_0 > 0.1$.

Until now, we only studied the case where all aggregate subunits interact with the condensate, corresponding to the binding parameter $\zeta = 1$. We now consider the case $\zeta = 0$, where aggregates interact exclusively with their ends with the condensate. The same quantities we studied before for $\zeta = 1$, i.e., the aggregation time, the aggregate mass and mass ratio, the total aggregate concentration, and the aggregate size ratio, are shown in Figs. 2, *D–F* and S1, *C–D*. For $\gamma > 1.5$, the time T_{99} decreases with mobility prefactor ξ_0 (see Fig. 2 *D*) and shows the same behavior as for $\zeta = 1$. For $\gamma < 1.5$, it initially decreases with ξ_0, but then starts to increase again. The aggregate mass ratio (see Fig. 2 *E*) initially increases with ξ_0, and decreases after reaching a maximum. It then stays constant with ξ_0, before once more decreasing. As a result, we can identify four different regimes. The same regimes can also be found in the total aggregate number concentration Fig. 2 *F*, which shows a nonmonotonous dependence on ξ_0.

Switch-like transition in the aggregation dynamics upon changes in condensate rheology

In the previous section, we showed that there is transition between different regimes in the aggregate mass ratio (see Fig. 2, *B* and *E*) as a function of the values of the mobility exponent γ and prefactor ξ_0. In this section, we show that this transition can occur in a switch-like fashion. To see this behavior, which is reminiscent of a nonequilibrium bifurcation, we first compare the timescales of aggregate exchange between with the condensate and of aggregation. We show that the location of the transition in Fig. 2, *B* and *E* depends on whether aggregation is quicker than the aggregate exchange. Next, we numerically solve the master equation near this transition and find that the transition is indeed switch-like. The switch-like behavior is especially pronounced when all aggregate subunits interact with the condensate, $\zeta = 1$.

We first consider the time $T_{99}^{(\mathrm{mono})}$ of aggregation. This is the time it takes to assemble 99% of monomers in the case that only monomers are exchanged instantaneously between the inside and the outside of the condensate, while aggregates are not exchanged (see supporting material S1.2 for the analytical solution of this case). This corresponds to a high mobility exponent γ and mobility prefactor ξ_0. When comparing the aggregation time with the aggregate size-dependent exchange time τ_{f} (see Eq. 13), we find that only those aggregates with size i that fulfill $\tau_{\mathrm{f}} < T_{99}^{(\mathrm{mono})}$ are transported fast compared with the timescale of aggregation. For a given i, this corresponds to

$$\xi_0 > \frac{2V^{(\mathrm{I})}}{T_{99}^{(\mathrm{mono})}\left(\varphi^{(\mathrm{I})} - \varphi^{(\mathrm{II})}\right)\left(\chi_B - \chi_A\right)} i^{\gamma - \zeta}, \tag{16}$$

where we combined Eqs. 10 and 13 and supporting material S1.2.

Properties of switch-like transition when all aggregate subunits interact with condensate ($\zeta = 1$)

For $\zeta = 1$, linear aggregates are dominantly transported from phase II to I, since the partition coefficient P_i increases exponentially with aggregate size i (see Eq. 10). We now show that, with the help of the relation given in Eq. 16, we can predict the location of the transition between the two regimes of the mass ratio in Fig. 2 *B*. Then, we show that the mass ratio changes dramatically over multiple orders of magnitude for small changes of the mobility exponent γ, which we interpret as a switch-like behavior.

During the time of aggregation, for the most part, aggregates of size i that fulfill $T_{99}^{(\mathrm{mono})} > \tau_\mathrm{f}$ are transported between the two phases. In Fig. 2 *B*, we show blue curves that fulfill $T_{99}^{(\mathrm{mono})} = \tau_\mathrm{f}$ for different values i and we find that, for a certain range of size i, we can qualitatively predict where the transition between the two regions in Fig. 2 *B* happens. This implies that, for small values of γ or high values of ξ_0, aggregates of all sizes are exchanged between the inside and outside of the condensate, explaining the extremely high mass ratio in Fig. 2 *B*. For high values of γ or small ξ_0, the relation in Eq. 16 is only fulfilled for small aggregate sizes i, hence only monomers and small linear aggregates are exchanged, reducing the mass ratio and affecting the aggregation within phase I. For small values of both the mobility coefficient ξ_0 and the mobility coefficient $\gamma < 1$, monomers are exchanged slower than aggregates grow and as a result, the aggregation time is slower than in the homogeneous case (see Fig. 2 *A*). This difference is because aggregates that are generated in phase II are transported into phase I, while monomers remain in phase II. Within phase I, secondary nucleation and monomer pickup are slowed down since monomers are depleted.

Equation 16 allows us to derive a characteristic aggregate size for which material exchange happens faster than aggregation. However, it is important to note that the mean aggregate size $M_A^{(\alpha)}/c_A^{(\alpha)}$ is not constant during aggregation, but time dependent. To get an estimate of a characteristic aggregate size, we determine the maximal aggregate size. Therefore, we study the aggregate concentrations for the homogeneous aggregation (see supporting material S1.1 and Fig. S2) and find that the aggregate profile possesses a moving front. This front originates from the finite time the monomers have to aggregate. Aggregates with a size exceeding the front did not have sufficient time to assemble. The highest value of this front has the same order of magnitude as the aggregate size for which Eq. 16 best recovers the location of the transition in Fig. 2 *B*), $i \approx 10^4 - 10^5$. This observation suggests that the transition in the aggregate mass ratio (see Fig. 2 *B*) happens when all linear aggregates that exist in the system are exchanged faster between the condensate interior and exterior than the aggregation dynamics. We are now able to explain the two regimes (for $\xi_0 > 1\ \mu\mathrm{m}^3/\mathrm{s}$) found in Fig. 2 *B*, characterized by significant differences in the aggregate mass ratio. We conclude that, in the region defined by small mobility prefactor ξ_0 or large enough mobility exponent γ, monomers and small aggregates are dominantly exchanged between the two phases, since the timescale of aggregate exchange is too large compared with the time of aggregation for larger aggregates. Hence, aggregates remain outside of the condensate, decreasing the mass ratio. When the mobility exponent γ is reduced or the mobility prefactor ξ_0 is increased, the timescale of aggregate exchange deceases until the largest aggregates can be exchanged on a timescale faster than aggregation. This defines the/a second region for low values of γ or high ξ_0 in the phase diagram, which is characterized by a significantly increased aggregate mass ratio since all aggregates are transported into phase I.

For $\xi_0 < 1\ \mu\mathrm{m}^3/\mathrm{s}$, we also discover another transition that is linked to whether oligomers of size $i = 2$ are exchanged between the phases. For values of ξ_0 that are smaller than the the transition marked by the blue dashed line in Fig. 2 *B*, oligomers are not exchanged while aggregation is happening.

We next investigate the behavior near the transition. In Fig. 3, *A* and *B*, we show the aggregate mass ratio as a function of the mobility exponent γ and mobility prefactor ξ_0. The mass ratio shows a pronounced change with small changes of the mobility, reminiscent of a switch between two different limits. The position of the peak is increasing with the mobility prefactor ξ_0 and exponent γ. This suggests that, by varying its rheological features slightly, a cell can alter the qualitative outcome of the aggregate distribution near the region where the transition happens. Significant changes of behavior take place in a continuous manner for the mobility prefactor ξ_0. For the exponent γ, significant changes take place for transitions between the different regimes (Zimm, Rouse, and reptation regime).

For $\zeta = 1$, the outcome of the aggregation has two limits that we can investigate analytically (see supporting material S1.2): for small values of γ and high values of ξ_0, aggregates and monomers are exchanged instantaneously (see supporting material S1.2.2 for the analytical solution). Since the partition coefficient for aggregates, P_i, increases with size for $\zeta = 1$, aggregates that form outside the condensate are transported into the condensate. For high enough values of γ, only monomers are exchanged, while aggregates remain outside or inside of the condensate, depending on where they have formed (see supporting material S1.2.1 for the analytical solution). In Fig. S3, we solve the master equations in these limits numerically and show the excellent agreement with the analytical predictions.

Properties of switch-like transition when only aggregate ends interact with condensate ($\zeta = 0$)

We next investigate the case where only the ends of linear aggregates interact with the condensate (binding parameter $\zeta = 0$). In this case, the partition coefficient P_i is independent of aggregate size i and a substantial amount of long aggregates is located outside of the condensates since P_i is considerably smaller than in the previous case with $\zeta = 1$. Monomers and short linear aggregates form larger aggregates within phase I, leading to an influx from phase II to I of monomers. As a result, aggregates dominantly form within the condensate. The resulting difference in chemical potentials leads to an outflux of aggregates from phase I to II. First, we again use the relation in Eq. 16 to predict the location of the transition in the mass ratio between the inside and outside of the condensate (see Fig. 2 *E*). Then, we investigate and find that, near the transition, small changes in the

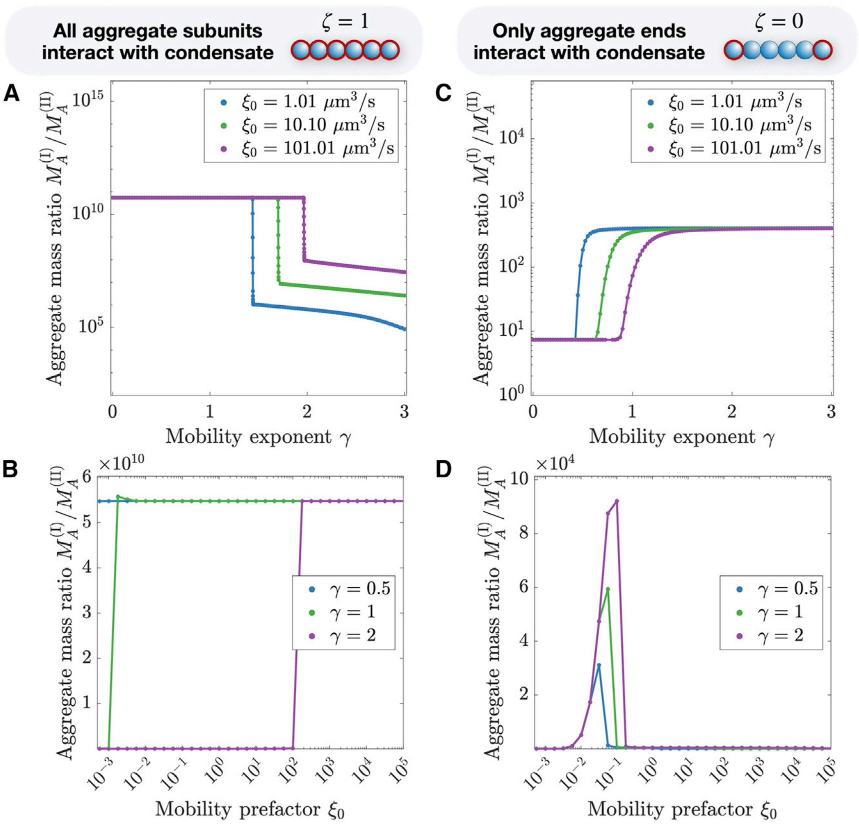

FIGURE 3 Switch-like transition between different rheology-dependent behaviors of the aggregate mass ratio. For the binding parameter $\zeta = 1$ and $\zeta = 0$, we show the aggregate mass ratio as a function of the aggregate mobility exponent γ (*A* and *C*) and for different values of the mobility prefactor ξ_0 (*B* and *D*) (see Eq. 12). The pronounced change of the mass ratio for small variations of the mobility over multiple orders of magnitudes is reminiscent of a nonequilibrium bifurcation. The total initial monomer concentration is $c_1^{(\mathrm{tot})}(t = 0) = 4\ \mu\mathrm{M}$ (see Eq. 14) and the volumes are $V^{(\mathrm{I})} = 10.1\ \mu\mathrm{m}^3$ and $V^{(\mathrm{II})} = 1000\ \mu\mathrm{m}^3$. For (*A*) and (*B*) we used $P_i = \exp(2i)$ and for (*C*) and (*D*) we used $P_i = \exp(2)$. To see this figure in color, go online.

mobility exponent γ still lead to big changes in the mass ratio, but less dramatically than for $\zeta = 1$.

By comparing the aggregation time and the exchange time τ_f, we can predict when exchange of aggregates of size i happens on the same timescale as the aggregation for the case of exclusive and instantaneous monomer partitioning $T_{99}^{(\mathrm{mono})}$ (see *blue lines* in Fig. 2 *E*). For aggregate sizes comparable with the front position in the homogeneous case, corresponding to the largest aggregates observed in that case (see Fig. S2), we can recover the location of the observed transition in diagrams (see *blue curves* in Fig. 2 *E*). In addition, we observe another transition for values around $\xi_0 = 0.01 - 0.1\ \mu\mathrm{m}^3/\mathrm{s}$ that can be linked to the exchange of oligomers, $i = 2$.

We next investigate the dependence of the aggregate mass ratio on the mobility exponent γ. Analogously to the case with $\zeta = 1$, we find two distinct regions in Fig. 2 *E* for $\xi_0 > 1\ \mu\mathrm{m}^3/\mathrm{s}$. By investigating this transition (see Fig. 3, *C* and *D*), we find that the transitions happens over a larger range of values of γ and ξ_0 than for $\zeta = 1$. Interestingly, for changes of γ, the direction of the transition is opposite to the one observed for $\zeta = 1$, i.e., increasing the mobility exponent γ leads to an increase of the mass ratio. By altering how the condensate is interacting with the linear aggregates, a cell is capable of dramatically altering the outcome of the aggregate distribution in the presence of liquid condensates. For $\xi_0 < 1\ \mu\mathrm{m}^3/\mathrm{s}$, we discover another pronounced transition that is resulting from oligomer exchange (with $i = 2$).

Even though the transition for $\xi_0 > 1\ \mu\mathrm{m}^3/\mathrm{s}$ is more smooth, we can still consider two limits between which the transition takes place: For large enough values of γ and small enough values of ξ_0, we expect to observe exclusively monomer exchange (see supporting material S1.2.1 for the analytical solution). Importantly, this means that the mass ratio, a quantity that can be accessed experimentally by fluorescently tagging the aggregates and monomers, can exceed the aggregate partition coefficient, that was here chosen to be $P_i = \exp(2)$, but reaches values up to 10^4 for the numerical solutions of the master equations. It is important to note that this results from the rheological features of the condensate. For small values of γ and high enough values of ξ_0, all aggregates are rapidly exchanged between both phases and the mass ratio is identical to the partition coefficient (see supporting material S1.2.2 for the analytical solution). But since the partition coefficient is now not dependent on the aggregate size, aggregates generated in phase I will be transported to phase II to fulfill the partition coefficient P_i and the aggregate mass ratio will be smaller and reach P_i. This is also consistent with the aggregation time that increases for small γ (see Fig. 2 *D*) since the concentration of aggregation within I is decreased, reducing the effects of secondary nucleation and linear aggregate elongation (see Fig. 1 *A*). This has also an effect on the total

aggregate concentration (see Fig. 2 *F*), resulting in a lower aggregate concentration for small values of γ. We numerically solve the master equations in both limits and find excellent agreement with the analytical predictions (see Fig. S3 and supporting material S1.2).

Linear aggregation kinetics in a condensate with complex rheological properties

In the previous section, we considered the case where the aggregate mobility ξ_i is governed by a single power law with the exponent γ. If linear aggregates are embedded in a network of phase-separating molecules A and B, we need to account for diffusion of linear aggregates in a complex liquid environment. For example, condensates composed of reconstituted proteins in vitro were shown to exhibit a viscoelastic rheology consistent with a Maxwell-like model (35,52). Moreover, it has been reported that phase-separated biopolymer melts can have pore sizes in the order of 10 nm (53,54).

A simple model for such complex rheological properties takes into account that the diffusion coefficient follows mixed scalings as a function of aggregate size with multiple crossovers. Here, we study two scaling regimes with one crossover, which may present a simple model for a condensate where the phase-separating components form pores of characteristic size. In this case, linear aggregates in a condensate follow a Rouse dynamics (mobility exponent $\gamma = 1$) when they are shorter than the pore size. When aggregates grow in length, they become entangled, for example with the material that forms the condensate. Then, aggregates diffuse similar to polymers in the reptation regime ($\gamma = 2$) (45,46,55). For this simple model with two scaling regimes, the mobility can be written as

$$\xi_i = \xi_0 \begin{cases} i^{-1}, & i < i_r \\ \left(i - i_r + \sqrt{i_r}\right)^{-2}, & i \geq i_r \end{cases}. \tag{17}$$

Here, i_r is the characteristic aggregate size where the transition from Rouse to reptation dynamics takes place, as shown in Fig. 4 *A*, and representing a measure of the pore size of the condensate. For $i_r = 1$, the mobility is identical to the reptation regime ($\gamma = 2$), in the limit $i_r \to \infty$ the mobility corresponds to the Rouse regime ($\gamma = 1$).

In Fig. 4 *B* we show the aggregate mass ratio for the case when aggregates bind over their whole length to the phase-separating material A and B, corresponding to the binding parameter $\zeta = 1$. Again, we observe a switch-like behavior, analogously to the behavior observed in Fig. 2 *B*, but now depending on the aggregate length i_r at which the transition from rouse to reptation dynamics happens instead of the mobility exponent γ. While for small enough values of i_r (for the chosen parameters $i_r < 10$), the behavior still corresponds to the reptation regime behavior. For $i_r > 10^4$, the behavior is again independent of i_r and corresponds to the Rouse-regime dynamics. This is in accordance with the maximal aggregate size in the system. In between, for $1 < i_r < 10^3$, we see a transition from the reptation regime-like behavior toward the Rouse-like behavior.

If aggregates only bind with their ends to A and B, $\zeta = 0$, we see that, for the chosen parameters in Fig. 4 *C*, the behavior is more complex: While again it is independent of the characteristic aggregate size for $i_r > 10^4$, for smaller values it undergoes a smooth transition from Rouse ($\gamma = 2$) to reptation dynamics ($\gamma = 1$).

We find that a switch-like transition can also occur for condensates of complex rheology by changes of the transition length i_r. By changing i_r, a cell can control at which

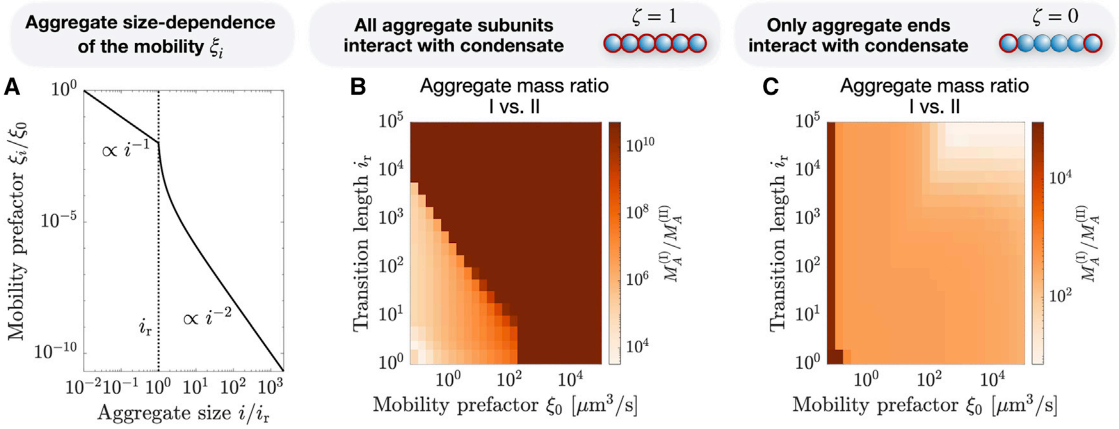

FIGURE 4 Linear aggregation dynamics for a more realistic dependence of the aggregate diffusion coefficient on aggregate size. (*A*) Plot of Eq. 17 with the transition length i_r measured in terms of monomer sizes and mobility prefactor ξ_0. (*B*) Aggregate mass ratio for $\zeta = 1$ with $P_i = \exp 2\, i$. For $i_r = 1$ the dynamics corresponds to the reptation regime ($\gamma = 2$)), for $i_r = \infty$ it corresponds to the Rouse regime ($\gamma = 1$)). We observe a transition between the Rouse and reptation dynamics around $i_r = 10 - 10^4$. (*C*) Aggregate mass ratio for $\zeta = 0$ with $P_i = \exp 2$. We observe a transition between the Rouse and reptation dynamics around $i_r = 1 - 10^3$. The total initial monomer concentration is $c_1^{(tot)}(t = 0) = 4\ \mu M$ (see Eq. 14) and the volumes are $V^{(I)} = 10.1\ \mu m^3$ and $V^{(II)} = 1000\ \mu m^3$. To see this figure in color, go online.

mobility the transition occurs. In other words, the characteristic pore size of the condensate material, which should be related to the transition length i_r, affects the aggregation kinetics. Specifically, bigger pores lead to Rouse dynamics with a higher aggregate mass ratio when all aggregate subunits interact with the condensate, $\zeta = 1$. If $\zeta = 0$, so that aggregates only interact with their ends with the condensate, bigger pores can lead to a smaller mass ratio, as long as the mobility prefactor is large enough.

To summarize, here we presented a numerical study that is as close as possible to experimentally tractable parameters. Our study enables a direct comparison to experimental systems. This comparision may provides us another way to ascertain how a cell can control linear aggregation.

CONCLUSION

We presented a theoretical framework to investigate how the kinetics of irreversible protein aggregation of dilute linear aggregates is affected by the rheological properties of phase-separated condensates. We found that condensate rheology can have a considerable impact on the overall aggregate distribution and aggregation kinetics. In particular, we discovered different regimes of monomer and aggregate transport between the inside and outside of the condensates (see Fig. 5, *A* and *B*): either only monomers are exchanged between the two phases on a timescale faster than the aggregation time, or both, monomers and long aggregates. In addition, we discovered that the way that linear aggregates interact with the phase-separating proteins can have a substantial impact on the distribution of aggregates and how fast monomers assemble (see Fig. 5, *A–C*). A key finding of our work is that slightly different rheological properties, reflected either in different mobility prefactor ξ_0 or mobility exponents γ (see Eq. 12), can lead to pronounced changes of the aggregate distribution. These pronounced changes occur in a very narrow window of controlling parameters indicating switch-like behavior. Due to irreversible kinetics, this transition is not a thermodynamic phase transition, rather the transition is reminiscent of a nonequilibrium bifurcation.

Our theoretical findings of a switch-like change of the aggregation kinetics due to changes in rheology can be experimentally scrutinized by in-vitro studies of droplets undergoing gelation or upon adding agents that affect viscosity. In living systems, various processes can cause changes in rheological properties (see Fig. 5 *D*). One example for such rheological changes are variations of the cytoplasmic concentration via an osmotic shift, which enables cells to alter the aggregation and disassembly of microtubules (56). Recently, it has been discovered that varying the pH level of the cell cytoplasm can mediate a transition between a fluid-like and a solid-like state, referred to as a dormant state (34,57). Thus, a cell possesses a large toolkit that it could use to alter its rheology and, as a result, the kinetics of physiological or aberrant aggregates. In the future, unraveling the link between intracellular changes in rheology and aggregation kinetics may pave the way to

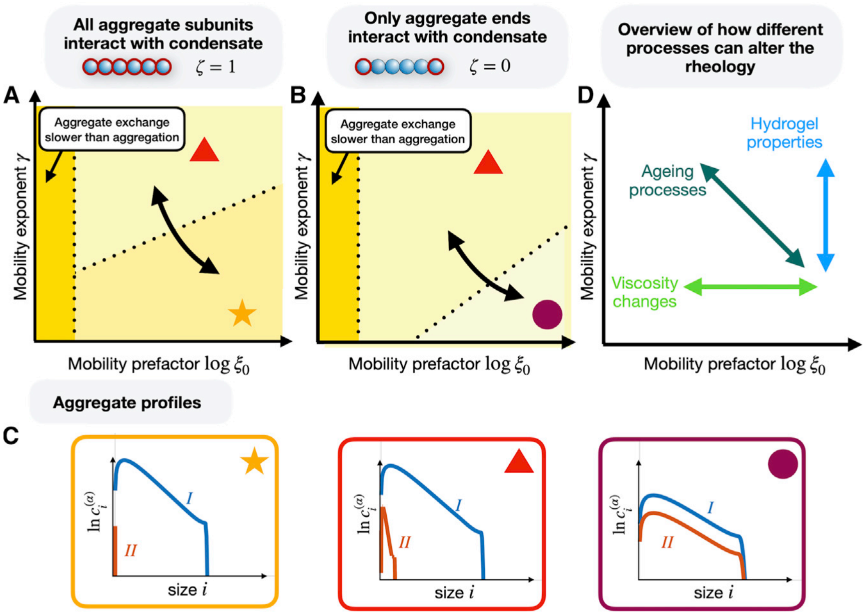

FIGURE 5 Overview of how condensate rheology can affect linear aggregation. We consider two cases: (*A*) either all aggregate subunits bind to the condensate, $\zeta = 1$, or (*B*) only aggregate ends bind to the condensate, $\zeta = 0$. In both cases, we discover different regimes that affect the aggregate size distribution, depending on the mobility exponent γ and prefactor ξ_0. For very small ξ_0, in both cases the exchange of monomers and aggregates is too slow and the aggregation in both phases is independent of each other. For higher values of the mobility prefactor ξ_0 and high values of the mobility exponent γ, in both cases we find that only monomers are exchanged between the compartments. We sketch the resulting aggregate distributions inside (phase I) and outside of the compartment (II) in (*C*) in the orange box and their location in (*A*) and (*B*) with the red triangle. For lower values of the mobility exponent γ, aggregates and monomers are exchanged between the inside and outside of the compartment. For $\zeta = 1$, this leads to an accumulation of most aggregates inside the condensate. This limit is shown via the orange star in (*A*) and (*C*). For $\zeta = 0$, the partition coefficient is constant and, while aggregates are exchanged, their ratio is fixed. In this case, the accumulation of aggregates inside the condensate is weaker and we can also find a nonnegligible amount of aggregates outside the condensate. In addition, the aggregate concentration follows $c_i^{(\mathrm{I})}/c_i^{(\mathrm{II})} = P_f$ with the fixed aggregate partitioning factor P_f. This limit is shown in (*B*) and (*C*) with the purple circle. In (*A*) and (*B*) we highlight with the black arrows in what direction a cell needs to change its rheology to transition from one limit to another one. In (*D*) we sketch how different physical processes can alter the rheology of a cellular condensate. To see this figure in color, go online.

understanding how aging or the transition to dormancy are linked to protein aggregation-related diseases.

Our theoretical model relies on a set of assumptions. For example, we assumed that linear aggregates are well-mixed inside and outside of the condensates at any time and that the flux between the condensate inside and outside is proportional to the chemical potential difference. However, while short aggregates homogenize quickly in each phase due to diffusion and very long aggregates are essentially not exchanged at all, aggregates of intermediate size are expected to transiently accumulate at the phase boundary contradicting a well-mixed assumption. In other words, the concentration of such aggregates may follow a nonlinear spatial profile around the interface that presumably decreases toward the center of the condensate. The nonlinear profile within the condensate will homogenize on a timescale that depends on the size of the condensate and aggregates diffusion coefficient. Future steps concern the derivation of a theory governing the time evolution of size distributions that vary in space. Such a theory enables investigation of the role of heterogeneous size distributions arising from a difference in the exchange flux between longer and shorter aggregates. The theory would also enable us to investigate the effect of spatially varying rheological properties. Importantly, we expect that, in most cases, such effects are only important close to the switch-like transition reported in this work. Another effect that we are ignoring in this study is that, in principle, aggregate size could exceed condensate size, potentially leading to kinks and ring-like arrangements of the linear aggregates (58) or to linear filaments emanating from the condensate (59). Capturing such effects also requires a spatial theory for the aggregation kinetics. In addition, aggregates in experimental systems are not necessarily dilute. Nondilute aggregation can affect phase separation of condensates. In this case, it is necessary to account for the feedback between phase separation and protein aggregation. Finally, we ignored aggregate fragmentation in this study, since it typically makes the solution of the governing equations significantly more complex. In the future, it will be interesting to study the role of fragmentation, using recently developed mathematical tools (60,61).

APPENDIX A. DETAILS OF NUMERICAL SOLUTION OF THE MASTER EQUATIONS AND ANALYSIS OF CONCENTRATION PROFILES

The numerical solution of the master equations (see Eqs. 1a and 1b) were performed on the local computing cluster of the MPI-PKS, consisting of X86-64 GNU/Linux systems. The code was written in C++. We used the GCC compiler (version 7.5). To numerically solve the master equations (see Eqs. 1a and 1b) we used a Euler algorithm to discretize time using a time step $\Delta t = 2$ ms.

The data were then analyzed with custom scripts written in MATLAB R2020b (The MathWorks, Natick, MA). The moving front in Fig. S3, *J–L* is identified by first normalizing the concentration profiles

$$\tilde{c}_i^{(\alpha)} = \frac{c_i^{(\alpha)}}{\sum_{i=1}^{M} c_i^{(\alpha)}} \tag{18}$$

and then only picking the region where $i > 300$ and $\tilde{c}_i^{(\alpha)} > 10^{-20}$. We then compute the logarithm of the concentrations $\tilde{c}_i^{(\alpha)}$ and compute the 100-mean average with the MATLAB function movmean(). We find peaks of this curve, corresponding to region with a high concentration gradient, with the function findpeaks() and choose the one that corresponds to the smallest value of i.

APPENDIX B. VOLUME FRACTION OF AMYLOID MONOMERS AND FIBRILS

The physiological concentration of amyloid-β monomers is around $c_1 = 90 - 150$ pM (50,51). The average hydrodynamic radius of amyloid-β is around $R_1 = 1 - 3$ nm (63–65). The corresponding monomer volume is given by $V_1 = \frac{4}{3}\pi R_1^3$. The volume fraction then results from

$$\varphi_1 = c_1 N_A V_1 \sim 10^{-10} - 10^{-8}, \tag{19}$$

with the Avogadro constant $N_A = 6.022 \cdot 10^{23}$ mol^{-1}. Thus, the monomers are dilute.

The persistence length of amyloid-β fibrils of lengths between 0.2 and 5 μm is around $l_p \approx 4$ μm (66). For condensates that are smaller than the persistence length, we approximate aggregates as stiff rods. In that case, the volume of a linear aggregate consisting of i monomers is given by

$$V_i \simeq V_1 i. \tag{20}$$

When we consider that monomers of an initial concentration c_m are all exclusively assembled into aggregates of size i, the aggregates have a number concentration of

$$c_i = \frac{1}{i} c_1. \tag{21}$$

The volume fraction of aggregates consisting each of i monomers is then given by (using Eqs. 20, 21, and 19)

$$\varphi_i = c_i N_A V_i = c_1 N_A V_1 = \varphi_1. \tag{22}$$

This implies that if monomers are dilute, aggregates will be dilute too. Please note that for condensates that have a diameter smaller than the persistence length, $l_p \approx 4$ μm, the ends of aggregates are not necessarily located within the condensate. In this study, we are ignoring such effects and assume that the aggregates persistence length is smaller than the condensate diameter. If aggregate length is smaller than condensate diameter, we can describe aggregates as spherical aggregates with a volume given by $V_i = \frac{4\pi}{3} R_g^3$. Here, we introduce the radius of gyration R_g^3. For a good solvent, the radius of gyration follows $R_g \simeq R_1 i^{\frac{3}{5}}$, for a bad solvent it follows $R_g \simeq R_1 i^{\frac{1}{3}}$ (45). Thus, the upper limit of the volume of a linear aggregate consisting of i monomers (for a good solvent) is given by

$$V_i \simeq V_1 i^{\frac{9}{5}}. \tag{23}$$

If all monomers with volume fraction φ_1 assemble exclusively into aggregates of length i, the volume fraction of the aggregates is then given by (using Eqs. 23, 21, and 19)

$$\varphi_i = c_i N_A V_i = i^{\frac{4}{5}} c_1 N_A V_1 = i^{\frac{4}{5}} \varphi_1. \tag{24}$$

Any linear aggregates assembled by dilute monomers are dilute as long as $i^{\frac{4}{5}} \ll 10^8$. This corresponds to amyloid aggregates consisting of 10^{10} subunits, which exceeds by several of orders the largest aggregates considered in our studies.

APPENDIX C. DERIVATION OF FREE ENERGY

Here, we consider an incompressible mixture of two phase-separating components A and B with monomers and linear aggregates of different sizes i. We assume that the monomers are highly diluted in respect to components of A and B. The total number $\Omega_i^{(\mathrm{tot})}$ of available states of N_i indistinguishable aggregates of size i is given by

$$\Omega_i^{(\mathrm{tot})} = \frac{1}{N_i!}\left(\frac{V}{v}\Omega_i^{(\mathrm{conf})}\right)^{N_i}. \tag{25}$$

Here, the term V/v corresponds to the total number of available locations for the aggregate centers with V the system volume and v the volume of a monomer. The second term $\Omega_i^{(\mathrm{conf})}$ corresponds to the internal configurations of the aggregates, which is for example affected by whether the aggregate is located in a good or poor solvent. Since the linear aggregates are dilute, we assume that each of them can be independently distributed in the volume V and there are no excluded volume effects, so that their internal configurations are not affected by the presence of other aggregates. In addition, we assume that the presence of components A and B has no effect on $\Omega_i^{(\mathrm{conf})}$. The mixing entropy for aggregates of size i then follows from

$$\begin{aligned} S_i &= k_\mathrm{B} \ln \Omega_i^{(\mathrm{tot})} \\ &= k_\mathrm{B}\left[N_i \ln\left(\frac{V}{v}\right) + N_i \ln \Omega_i^{(\mathrm{conf})} - N_i \ln N_i + N_i\right], \end{aligned} \tag{26}$$

where we have used Sterling's approximation $\ln(N!) \approx N \ln N - N$. The total mixing entropy, also taking into account the components A and B and aggregates of size $i \in \{1,\ldots,M\}$, is then given by

$$\begin{aligned} S_\mathrm{mix} = k_\mathrm{B}\frac{V}{v}\Bigg[& -\frac{\varphi_A}{n_A}\ln\varphi_A - \frac{\varphi_B}{n_B}\ln\varphi_B + \sum_{i=1}^{M}\left(\frac{\varphi_i}{i}\ln\Omega_i^{(\mathrm{conf})}\right. \\ & \left. - \frac{\varphi_i}{i}\ln\left(\frac{\varphi_i}{i}\right) + \frac{\varphi_i}{i}\right)\Bigg], \end{aligned} \tag{27}$$

with the linear aggregate volume fractions $\varphi i = ivNi/V$ of aggregates of size i, the component volume fractions φ_A and φ_B, with n_A and n_B, the nondimensional sizes of components A and B in multiples of v, and the maximal aggregate size M. Since we assume that A and B are not dilute, we can us to use the same mixing entropy contributions as in the Flory-Huggins theory.

Next to the mixing entropy, the enthalpy due to interactions of the aggregates with the components A and B also contributes to the free energy. For N_i aggregates of size i, the enthalpy is given by

$$\begin{aligned} H_i &= \Delta w_{i,A} i^{\zeta} N_i \varphi_A + \Delta w_{i,B} i^{\zeta} N_i \varphi_B \\ &= k_\mathrm{B}T\frac{V}{v}\left(\chi_{i,A} i^{\zeta-1}\varphi_i\varphi_A + \chi_{i,B} i^{\zeta-1}\varphi_i\varphi_B\right), \end{aligned} \tag{28}$$

where $\Delta w_{i,A}$ and $\Delta w_{i,B}$ are the change of energy per monomer of the linear aggregate in contact with components A and B and defining the interaction parameters $\chi_{i,A} = \Delta w_{i,A}/(k_\mathrm{B}T)$ and $\chi_{i,B} = \Delta w_{i,B}/(k_\mathrm{B}T)$. Here, the binding parameter ζ represents the limit of how the phase-separating components A and B interact with the aggregate: for $\zeta = 1$ over its full length and for $\zeta = 0$ only with the two linear aggregate ends (see Fig. 1 *C*). For $\zeta = 1$, the enthalpy term is identical to the one found for the Flory-Huggins theory since we can still make the mean field approximation for the components A and B.

The total enthalpy of the system is then given by

$$\begin{aligned} H = \sum_{i=1}^{M} H_i = k_\mathrm{B}T\frac{V}{v}\Bigg[& \chi\varphi_A\varphi_B + \sum_{i=1}^{M}\left(\chi_{i,A} i^{\zeta-1}\varphi_i\varphi_A\right. \\ & \left. + \chi_{i,B} i^{\zeta-1}\varphi_i\varphi_B\right)\Bigg], \end{aligned} \tag{29}$$

ignoring interactions of the aggregates with each other since we assume that they are far apart from each other and do not intersect. The complete free energy is then given by

$$\begin{aligned} F = k_\mathrm{B}T\frac{V}{v}\Bigg[& \frac{\varphi_A}{n_A}\ln\varphi_A + \frac{\varphi_B}{n_B}\ln\varphi_B - \sum_{i=1}^{M}\left(\frac{\varphi_i}{i}\ln\Omega_i^{(\mathrm{conf})}\right. \\ & \left. - \frac{\varphi_i}{i}\ln\left(\frac{\varphi_i}{i}\right) + \frac{\varphi_i}{i}\right) + \chi\varphi_A\varphi_B + \sum_{i=1}^{M}\left(\chi_{i,A} i^{\zeta-1}\varphi_i\varphi_A\right. \\ & \left. + \chi_{i,B} i^{\zeta-1}\varphi_i\varphi_B\right) + \sum_{i=1}^{M}\Delta w\varphi_i\frac{i-1}{i}\Bigg]. \end{aligned} \tag{30}$$

Here, we also introduced the bond energy Δw between each subunit pair bound to each other within an aggregate. For an aggregate of length i, there are $i-1$ subunits pairs. Since the linear aggregates are highly dilute, we can assume that the volume fractions of the components can be approximated by $\varphi_A = \varphi$ and $\varphi_B = 1 - \varphi - \sum_{j=1}^{M}\varphi_j$, so that the total free energy of the system reads

$$\begin{aligned} F \approx k_\mathrm{B}T\frac{V}{v}\Bigg[& \frac{\varphi}{n_A}\ln\varphi + \frac{\left(1-\varphi-\sum_{j=1}^{M}\varphi_j\right)}{n_B}\ln\left(1-\varphi\right. \\ & \left. - \sum_{j=1}^{M}\varphi_j\right) - \sum_{i=1}^{M}\left(\frac{\varphi_i}{i}\ln\Omega_i^{(\mathrm{conf})} - \frac{\varphi_i}{i}\ln\left(\frac{\varphi_i}{i}\right) + \frac{\varphi_i}{i}\right) \\ & + \chi\varphi\left(1-\varphi-\sum_{j=1}^{M}\varphi_j\right) + \sum_{i=1}^{M}\left(\chi_{i,A} i^{\zeta-1}\varphi_i\varphi\right. \\ & \left. + \chi_{i,B} i^{\zeta-1}\varphi_i(1-\varphi)\right) + \sum_{i=1}^{M}\Delta w\varphi_i\frac{i-1}{i}\Bigg]. \end{aligned} \tag{31}$$

APPENDIX D. AGGREGATION KINETICS IN COEXISTING PHASES

For simplicity, we will now consider the special case of monomer pickup and investigate how phase equilibrium affects the aggregation dynamics.

The calculations are based on (37). The chemical potential given in Eq. 7 can be written as

$$\mu_i^{(\alpha)} = k_B T\left[\ln\left(\frac{\varphi_i}{i}\right) + i\Lambda^{(\alpha)} + \ln \Omega_i^{(\text{conf})} + \Delta w(i-1)\right]. \tag{32}$$

We introduce the phase-dependent term ($\alpha = \text{I}, \text{II}$)

$$\Lambda^{(\alpha)} = -\frac{\ln\left(1-\varphi^{(\alpha)}\right)}{n_B} - \frac{1}{n_B} + i^{\zeta-1}\varphi^{(\alpha)}\chi_A + i^{\zeta-1}\left(1-\varphi^{(\alpha)}\right)\chi_B - \chi\varphi^{(\alpha)}. \tag{33}$$

For monomer pickup (see Fig. 1 *A*), we consider an irreversible process with the rate

$$r_+^{(\alpha)} = \tilde{k}_+^{(\alpha)} a_1^{(\alpha)} a_i^{(\alpha)}. \tag{34}$$

Here, we introduce the chemical activities $a_i^{(\alpha)} = \gamma_i^{(\alpha)} c_i^{(\alpha)}$ with the activity coefficients $\gamma_i^{(\alpha)}$. The rate can be written as

$$\begin{aligned} r_+^{(\alpha)} &= \tilde{k}_+^{(\alpha)} \exp\left[\frac{\mu_1+\mu_i}{k_B T}\right] \\ &= \tilde{k}_+^{(\alpha)} c_1^{(\alpha)} c_i^{(\alpha)} \exp\left[\Lambda^{(\alpha)}(i+1) + \ln \Omega_i^{(\text{conf})} + \Delta w(i-1)\right]\nu^2. \end{aligned} \tag{35}$$

where we use a relationship between the activities and the chemical potentials of the form $\mu_i^{(\alpha)} = \ln(\gamma_i^{(\alpha)} c_i^{(\alpha)})$, which corresponds to the case of zero reference chemical potentials. The activity coefficients are

$$\gamma_i^{(\alpha)} = \exp\left[\Lambda^{(\alpha)} i + \ln \Omega_i^{(\text{conf})} + \Delta w(i-1)\right]\nu. \tag{36}$$

As shown in (37), the activity coefficients satisfy

$$\frac{\gamma_i^{(\text{I})}}{\gamma_i^{(\text{II})}} = \frac{1}{P_i} \tag{37}$$

at phase equilibrium.

The interaction parameters χ, χ_A, and χ_B typically have amplitudes of a few kBT (33,34), while the magnitude of the bond energy Δw is much bigger since we consider irreversible aggregation. In this case, we can approximately write

$$r_+^{(\alpha)} \approx \tilde{k}_+^{(\alpha)}(i) c_1^{(\alpha)} c_i^{(\alpha)} \exp\left[\ln \Omega_i^{(\text{conf})} + \Delta w(i-1)\right]\nu^2. \tag{38}$$

When the coexisting phases are liquids, only the rate constants $k_+^{(\alpha)}$ and the concentrations depends on the respective phases. In the homogeneous case, the reaction rate reads

$$r_+ \approx \tilde{k}_+ c_1 c_i \exp\left[\ln \Omega_i^{(\text{conf})} + \Delta w(i-1)\right]\nu^2. \tag{39}$$

According to previous experimental studies of homogeneous systems (14,15,17), there is no significant dependence of the reaction rate on aggregate length i, i.e.,

$$r_+ \approx k_+ c_1 c_i. \tag{40}$$

To be consistent with these experimental measurements, we choose the reaction rate constant $\tilde{k}_+(i) \propto \exp[-\ln \Omega_i^{(\text{conf})} - \Delta w(i-1)]$. Analogously, we obtain for the case with two phases

$$r_+^{(\alpha)} \approx k_+^{(\alpha)} c_1^{(\alpha)} c_i^{(\alpha)}. \tag{41}$$

Analog steps can be performed for the nucleation processes, finally leading to Eqs. 1a and 1b.

APPENDIX E. RELATIONSHIP BETWEEN AGGREGATE MOBILITY AND AGGREGATE DIFFUSION COEFFICIENT

To derive the relation between the mobility coefficient ξ_i of aggregates of size i (see Eq. 12) and the corresponding diffusion coefficient D_i, we consider weak concentration profiles of the concentrations of aggregates. In this case, the mobility in each phase is approximately constant. Moreover, we focus on the limiting case where the reaction kinetics is slower than the exchange kinetics between the phases, suggesting a quasistatic approximation for the diffusive exchange between the phases. For such quasistatic conditions, the concentration profile is determined by the Laplace equation,

$$\Delta c_i^{(\alpha)}(r,t) = 0, \tag{42}$$

where time characterizes the slow transient due to the aggregation kinetics. For simplicity, we solve the Laplace equation for small spherical condensed phases with radius R in a finite systems of size L, where $R \ll L$. For slow reaction steps and small enough condensates, concentration gradients within the condensate $c_i^{(\text{I})}(r,t)$ can be neglected compare to the surrounding phase, i.e.,

$$c_i^{(\text{I})}(r,t) = c_i^{(\text{I})}(t). \tag{43}$$

For the surrounding phase II, we find approximately (approximation refers to neglecting terms of the order R/L)

$$c_i^{(\text{II})}(r,t) \simeq c_i^{(\text{II})}(L,t) + \frac{R}{r}\left[c_i^{(\text{II})}(R,t) - c_i^{(\text{II})}(L,t)\right], \tag{44}$$

where $c_i^{(\text{II})}(R,t)$ denotes the concentration directly at the interface of phase II. Applying partition equilibrium, we obtain

$$c_i^{(\text{II})}(r,t) = c_i^{(\text{II})}(L,t) + \frac{R}{r}\left[c_i^{(\text{I})}(t)\Big/P_i - c_i^{(\text{II})}(L,t)\right]. \tag{45}$$

Next, the radial flux $\vec{j}_i = j_i\vec{e}_r$ is given by

$$j_i = -D_i\partial_r c_i, \tag{46}$$

where D_i denotes the diffusion coefficient of aggregate of size i. At the interface $r = R$, the radial flux reads

$$j_i = \frac{D_i}{R}\left[c_i^{(\text{I})}(t)\Big/P_i - c_i^{(\text{II})}(L,t)\right]. \tag{47}$$

The total exchange rate J_i (units: 1/s) (Eq. 11) is related to the radial flux by $J_i = 4\pi R^2 j_i$ leading to

$$J_i = 4\pi R D_i \left[c_i^{(I)}(t) \Big/ P_i - c_i^{(II)}(L,t) \right]. \tag{48}$$

Comparing with linearization of Eq. 11 (see also (33)), we find the following relationship between the diffusion coefficient and the mobility:

$$\xi_i = 4\pi R D_i. \tag{49}$$

This relationships allows us to estimate the range of diffusion coefficients that correspond to the range of mobilities considered in our work. Please note that this estimate depends on the condensate radius R. We consider typical values of condensate radii, i.e., $R = \{0.1, 1, 10\}$ μm. For amyloid monomers, the reported diffusion coefficient is around $D_0 \approx 100$ $\mu m^2/s$ (65). For the condensate radii given above, the corresponding mobilities are $\xi_0 = \{10^2, 10^3, 10^4\}$ $\mu m^3/s$. In our simulations, we consider mobility values in this range as well as a bit higher and lower for the sake of generality. Thus, we probe experimentally relevant parameter values for the mobilities.

APPENDIX F. CHARACTERISTIC TIME OF LINEAR AGGREGATE EXCHANGE BETWEEN PHASES

In Eq. 11, we give the flux J_i of linear aggregates of size i between two coexisting phases I and II. Assuming that $c_i^{(I)}/c_i^{(II)} < P_i$, the dynamic equations of the aggregate concentration are written as

$$\frac{dc_i^{(I)}}{dt} = -\frac{J_i}{V^{(I)}} = -\frac{\xi_i\left(c_i^{(I)} + c_i^{(II)}\right)}{2V^{(I)}}\left[\ln\left(\frac{c_i^{(I)}}{c_i^{(II)}}\right) - \ln P_i\right], \tag{50a}$$

$$\frac{dc_i^{(II)}}{dt} = \frac{J_i}{V^{(II)}} = \frac{\xi_i\left(c_i^{(I)} + c_i^{(II)}\right)}{2V^{(II)}}\left[\ln\left(\frac{c_i^{(I)}}{c_i^{(II)}}\right) - \ln P_i\right], \tag{50b}$$

with the partition coefficient P_i. In our system, we usually find $c_i^{(I)} \geq c_i^{(II)}$. This implies that $\ln(c_i^{(I)}/c_i^{(II)}) \geq 0$. If $c_i^{(I)}/c_i^{(II)} \ll P_i$ we can then simplify the equations and get

$$\frac{dc_i^{(I)}}{dt} \simeq \frac{\xi_i\left(c_i^{(I)} + c_i^{(II)}\right)}{2V^{(I)}}\ln P_i, \tag{51a}$$

$$\frac{dc_i^{(II)}}{dt} \simeq -\frac{\xi_i\left(c_i^{(I)} + c_i^{(II)}\right)}{2V^{(II)}}\ln P_i. \tag{51b}$$

The solution of the equations has a single characteristic timescale

$$\tau_f = \frac{2V^{(I)}V^{(II)}}{\xi_i \ln P_i\left(V^{(II)} - V^{(I)}\right)}. \tag{52}$$

For $V^{(II)} \gg V^{(I)}$, we find

$$\tau_f = \frac{2V^{(I)}}{\xi_i \ln P_i}. \tag{53}$$

SUPPORTING MATERIAL

Supporting material can be found online at https://doi.org/10.1016/j.bpj.2022.11.009.

AUTHOR CONTRIBUTIONS

W.P., T.C.T.M., and C.A.W. designed the research. W.P. carried out all numerical solutions. W.P. and T.C.T.M. carried out analytical calculations. W.P. analyzed the data. W.P., T.C.T.M., and C.A.W. wrote the article.

ACKNOWLEDGMENTS

We thank Giacomo Bartolucci, Sudarshana Laha, Jonathan Bauermann, Guillaume Salbreux, and the Salbreux Lab for helpful discussions. W.P. kindly acknowledges financial support from the Herchel Smith Fund (Herchel Smith Postdoctoral Fellowship). C.A.W. acknowledges the financial support by the European Research Council under the European Union's Horizon 2020 research and innovation program (grant agreement no. 949021, "FuelledLife").

DECLARATION OF INTERESTS

The authors declare no competing interests.

REFERENCES

1. Taglialegna, A., I. Lasa, and J. Valle. 2016. Amyloid structures as biofilm matrix scaffolds. *J. Bacteriol.* 198:2579–2588.
2. Andreasen, M., G. Meisl, …, T. P. J. Knowles. 2019. Physical determinants of amyloid assembly in biofilm formation. *mBio.* 10:022799-18.
3. Oosawa, F., and S. Asakura. 1975. Thermodynamics of the Polymerization of Protein. Academic Press.
4. Steinmetz, M. O., K. N. Goldie, and U. Aebi. 1997. A correlative analysis of actin filament assembly, structure, and dynamics. *J. Cell Biol.* 138:559–574.
5. Salbreux, G., G. Charras, and E. Paluch. 2012. Actin cortex mechanics and cellular morphogenesis. *Trends Cell Biol.* 22:536–545.
6. Dobson, C. M. 2003. Protein folding and misfolding. *Nature.* 426:884–890.
7. Hardy, J., and D. J. Selkoe. 2002. The amyloid hypothesis of Alzheimer's disease: progress and problems on the road to therapeutics. *Science.* 297:353–356.
8. Walsh, D. M., and D. J. Selkoe. 2020. Amyloid β-protein and beyond: the path forward in Alzheimer's disease. *Curr. Opin. Neurobiol.* 61:116–124.
9. Iadanza, M. G., M. P. Jackson, …, S. E. Radford. 2018. A new era for understanding amyloid structures and disease. *Nat. Rev. Mol. Cell Biol.* 19:755–773.
10. Oosawa, F., and M. Kasai. 1962. A theory of linear and helical aggregations of macromolecules. *J. Mol. Biol.* 4:10–21.
11. Ferrone, F. A., J. Hofrichter, and W. A. Eaton. 1985. Kinetics of sickle hemoglobin polymerization: I. Studies using temperature-jump and laser photolysis techniques. *J. Mol. Biol.* 183:591–610.

12. Ferrone, F. A., J. Hofrichter, and W. A. Eaton. 1985. Kinetics of sickle hemoglobin polymerization: II. A double nucleation mechanism. *J. Mol. Biol.* 183:611–631.

13. Knowles, T. P. J., C. A. Waudby, …, C. M. Dobson. 2009. An analytical solution to the kinetics of breakable filament assembly. *Science.* 326:1533–1537.

14. Michaels, T. C. T., G. A. Garcia, and T. P. J. Knowles. 2014. Asymptotic solutions of the Oosawa model for the length distribution of biofilaments. *J. Chem. Phys.* 140, 194906.

15. Michaels, T. C. T., S. I. A. Cohen, …, T. P. J. Knowles. 2016. Hamiltonian dynamics of protein filament formation. *Phys. Rev. Lett.* 116, 038101.

16. Dayeh, M. A., G. Livadiotis, and S. Elaydi. 2018. A discrete mathematical model for the aggregation of β-amyloid. *PLoS One.* 13, e0196402.

17. Michaels, T. C. T., A. Šarić, …, T. P. J. Knowles. 2018. Chemical kinetics for bridging molecular mechanisms and macroscopic measurements of amyloid fibril formation. *Annu. Rev. Phys. Chem.* 69:273–298.

18. Brangwynne, C. P., C. R. Eckmann, …, A. A. Hyman. 2009. Germline P granules are liquid droplets that localize by controlled dissolution/condensation. *Science.* 324:1729–1732.

19. Hyman, A. A., C. A. Weber, and F. Jülicher. 2014. Liquid-liquid phase separation in biology. *Annu. Rev. Cell Dev. Biol.* 30:39–58.

20. Banani, S. F., H. O. Lee, …, M. K. Rosen. 2017. Biomolecular condensates: organizers of cellular biochemistry. *Nat. Rev. Mol. Cell Biol.* 18:285–298.

21. Patel, A., H. O. Lee, …, S. Alberti. 2015. A liquid-to-solid phase transition of the ALS protein FUS accelerated by disease mutation. *Cell.* 162:1066–1077.

22. Molliex, A., J. Temirov, …, J. P. Taylor. 2015. Phase separation by low complexity domains promotes stress granule assembly and drives pathological fibrillization. *Cell.* 163:123–133.

23. Alberti, S., and A. A. Hyman. 2021. Biomolecular condensates at the nexus of cellular stress, protein aggregation disease and ageing. *Nat. Rev. Mol. Cell Biol.* 1–18. https://doi.org/10.1038/s41580-020-00326-6.

24. Woodruff, J. B., B. Ferreira Gomes, …, A. A. Hyman. 2017. The centrosome is a selective condensate that nucleates microtubules by concentrating tubulin. *Cell.* 169:1066–1077.e10.

25. Mateju, D., T. M. Franzmann, …, S. Alberti. 2017. An aberrant phase transition of stress granules triggered by misfolded protein and prevented by chaperone function. *EMBO J.* 36:1669–1687.

26. Wei, M.-T., Y.-C. Chang, …, C. P. Brangwynne. 2020. Nucleated transcriptional condensates amplify gene expression. *Nat. Cell Biol.* 22:1187–1196.

27. Klein, I. A., A. Boija, …, R. A. Young. 2020. Partitioning of cancer therapeutics in nuclear condensates. *Science.* 368:1386–1392.

28. Agarwal, S., M. A. Klocke, …, E. Franco. 2021. Dynamic self-assembly of compartmentalized DNA nanotubes. *Nat. Commun.* 12:3557.

29. Li, P., S. Banjade, …, M. K. Rosen. 2012. Phase transitions in the assembly of multivalent signalling proteins. *Nature.* 483:336–340.

30. McCall, P. M., S. Srivastava, …, M. V. Tirrell. 2018. Partitioning and enhanced self-assembly of actin in polypeptide coacervates. *Biophys. J.* 114:1636–1645.

31. Wiegand, T., and A. A. Hyman. 2020. Drops and fibers—how biomolecular condensates and cytoskeletal filaments influence each other. *Emerg. Top. Life Sci.* 4:247–261.

32. Küffner, A. M., M. Linsenmeier, …, P. Arosio. 2021. Sequestration within biomolecular condensates inhibits Aβ-42 amyloid formation. *Chem. Sci.* https://doi.org/10.1039/D0SC04395H.

33. Weber, C., T. Michaels, and L. Mahadevan. 2019. Spatial control of irreversible protein aggregation. *Elife.* 8, e42315.

34. Adame-Arana, O., C. A. Weber, …, F. Jülicher. 2020. Liquid phase separation controlled by pH. *Biophys. J.* 119:1590–1605.

35. Jawerth, L., E. Fischer-Friedrich, …, F. Jülicher. 2020. Protein condensates as aging Maxwell fluids. *Science.* 370:1317–1323.

36. Cohen, S. I. A., M. Vendruscolo, …, T. P. J. Knowles. 2011. Nucleated polymerization with secondary pathways. I. Time evolution of the principal moments. *J. Chem. Phys.* 135, 065105.

37. Bauermann, J., S. Laha, …, C. A. Weber. 2021. Chemical kinetics and mass action in coexisting phases. Preprint at arXiv. https://doi.org/10.48550/arXiv.2112.07576.

38. Flory, P. J. 1942. Thermodynamics of high polymer solutions. *J. Chem. Phys.* 10:51–61.

39. Huggins, M. L. 1942. Some properties of solutions of long-chain compounds. *J. Phys. Chem.* 46:151–158.

40. Flory, P. J. 1953. Principles of Polymer Chemistry. Cornell University Press.

41. Kaddour, L. O., M. S. Anasagasti, and C. Strazielle. 1987. Molecular weight dependence of interaction parameter and demixing concentration in polymer-polymer-good solvent systems. comparison with theory. *Makromol. Chem.* 188:2223–2230.

42. Crawford, N., and M. D. Dadmun. 2006. The effect of polymer chain length on the thermodynamics of acrylate/cyanobiphenyl mixtures. *Liq. Cryst.* 33:195–203.

43. Smedes, F., R. W. Geertsma, …, K. Booij. 2009. Polymer- water partition coefficients of hydrophobic compounds for passive sampling: application of cosolvent models for validation. *Environ. Sci. Technol.* 43:7047–7054.

44. Teraoka, I. 1996. Polymer solutions in confining geometries. *Prog. Polym. Sci.* 21:89–149.

45. Doi, M., and S. F. Edwards. 1988. The Theory of Polymer Dynamics73. Oxford University Press.

46. Rubinstein, M., and R. H. Colby. 2003. Polymer Physics23. Oxford University Press.

47. Luby-Phelps, K. 2013. The physical chemistry of cytoplasm and its influence on cell function: an update. *Mol. Biol. Cell.* 24:2593–2596.

48. Sanders, D. W., N. Kedersha, …, C. P. Brangwynne. 2020. Competing protein-RNA interaction networks control multiphase intracellular organization. *Cell.* 181:306–324.e28.

49. Mogre, S. S., A. I. Brown, and E. F. Koslover. 2020. Getting around the cell: physical transport in the intracellular world. *Phys. Biol.* 17, 061003.

50. Lazarevic, V., S. Fieńko, …, A. Fejtova. 2017. Physiological concentrations of amyloid beta regulate recycling of synaptic vesicles via alpha7 acetylcholine receptor and CDK5/calcineurin signaling. *Front. Mol. Neurosci.* 10:221.

51. Waters, J. 2010. The concentration of soluble extracellular amyloid-β protein in acute brain slices from CRND8 mice. *PLoS One.* 5:e15709.

52. Jawerth, L. M., M. Ijavi, …, E. Fischer-Friedrich. 2020. Salt-dependent rheology and surface tension of protein condensates using optical traps. *Phys. Rev. Lett.* 125, 229901.

53. Frey, S., and D. Görlich. 2007. A saturated FG-repeat hydrogel can reproduce the permeability properties of nuclear pore complexes. *Cell.* 130:512–523.

54. Wei, M.-T., S. Elbaum-Garfinkle, …, C. P. Brangwynne. 2017. Phase behaviour of disordered proteins underlying low density and high permeability of liquid organelles. *Nat. Chem.* 9:1118–1125.

55. Karatrantos, A., R. J. Composto, …, N. Clarke. 2019. Modeling of entangled polymer diffusion in melts and nanocomposites: a Review. *Polymers.* 11:876.

56. Molines, A. T., J. Lemière, …, F. Chang. 2020. Physical properties of the cytoplasm modulate the rates of microtubule growth and shrinkage. Preprint at bioRxiv. https://doi.org/10.1101/2020.10.27.352716.

57. Munder, M. C., D. Midtvedt, …, S. Alberti. 2016. A pH-driven transition of the cytoplasm from a fluid-to a solid-like state promotes entry into dormancy. *Elife.* 5, e09347.

58. Cohen, A. E., and L. Mahadevan. 2003. Kinks, rings, and rackets in filamentous structures. *Proc. Natl. Acad. Sci. USA.* 100:12141–12146.

59. Ray, S., N. Singh, …, S. K. Maji. 2020. α-Synuclein aggregation nucleates through liquid–liquid phase separation. *Nat. Chem.* 12:705–716.

60. Tournus, M., M. Escobedo, …, M. Doumic. 2021. Insights into the dynamic trajectories of protein filament division revealed by numerical investigation into the mathematical model of pure fragmentation. *PLoS Comput. Biol.* 17, e1008964.

61. Doumic, M., M. Escobedo, and M. Tournus. 2021. An inverse problem: recovering the fragmentation kernel from the short-time behaviour of the fragmentation equation. Preprint at arXiv. https://doi.org/10.48550/arXiv.2112.10423.

62. Meisl, G., X. Yang, …, T. P. J. Knowles. 2014. Differences in nucleation behavior underlie the contrasting aggregation kinetics of the Aβ40 and Aβ42 peptides. *Proc. Natl. Acad. Sci. USA.* 111:9384–9389.

63. Nag, S., B. Sarkar, …, S. Maiti. 2011. Nature of the amyloid-β monomer and the monomer-oligomer equilibrium. *J. Biol. Chem.* 286:13827–13833.

64. Zhang-Haagen, B., R. Biehl, …, D. Willbold. 2016. Monomeric amyloid beta peptide in hexafluoroisopropanol detected by small angle neutron scattering. *PLoS One.* 11:e0150267.

65. Novo, M., S. Freire, and W. Al-Soufi. 2018. Critical aggregation concentration for the formation of early Amyloid-β (1–42) oligomers. *Sci. Rep.* 8:1783–1788.

66. vandenAkker, C. C., M. F. M. Engel, …, G. H. Koenderink. 2011. Morphology and persistence length of amyloid fibrils are correlated to peptide molecular structure. *J. Am. Chem. Soc.* 133:18030–18033.

Biophysical Journal, Volume *122*

Supplemental information

Aggregation controlled by condensate rheology

Wolfram Pönisch, Thomas C.T. Michaels, and Christoph A. Weber

Supporting Material

Wolfram Pönisch[1], Thomas C.T. Michaels[2,3,4], and Christoph A. Weber[5,6,7]

[1]Department of Physiology, Development and Neuroscience, University of Cambridge, Cambridge CB2 3DY, United Kingdom
[2]Department of Physics and Astronomy, Institute for the Physics of Living Systems, University College London, London, United Kingdom
[3]Institute of Biochemistry, ETH Zürich, Zürich, Switzerland
[4]Medical Research Council Laboratory for Molecular Cell Biology, University College London, London, United Kingdom
[5]Max Planck Institute for the Physics of Complex Systems, 01187 Dresden, Germany
[6]Center for Systems Biology Dresden, Pfotenhauerstrasse 108, 01307 Dresden, Germany
[7]Faculty of Mathematics, Natural Sciences, and Materials Engineering: Institute of Physics, University of Augsburg, Universitätsstr. 1, 86159 Augsburg, Germany

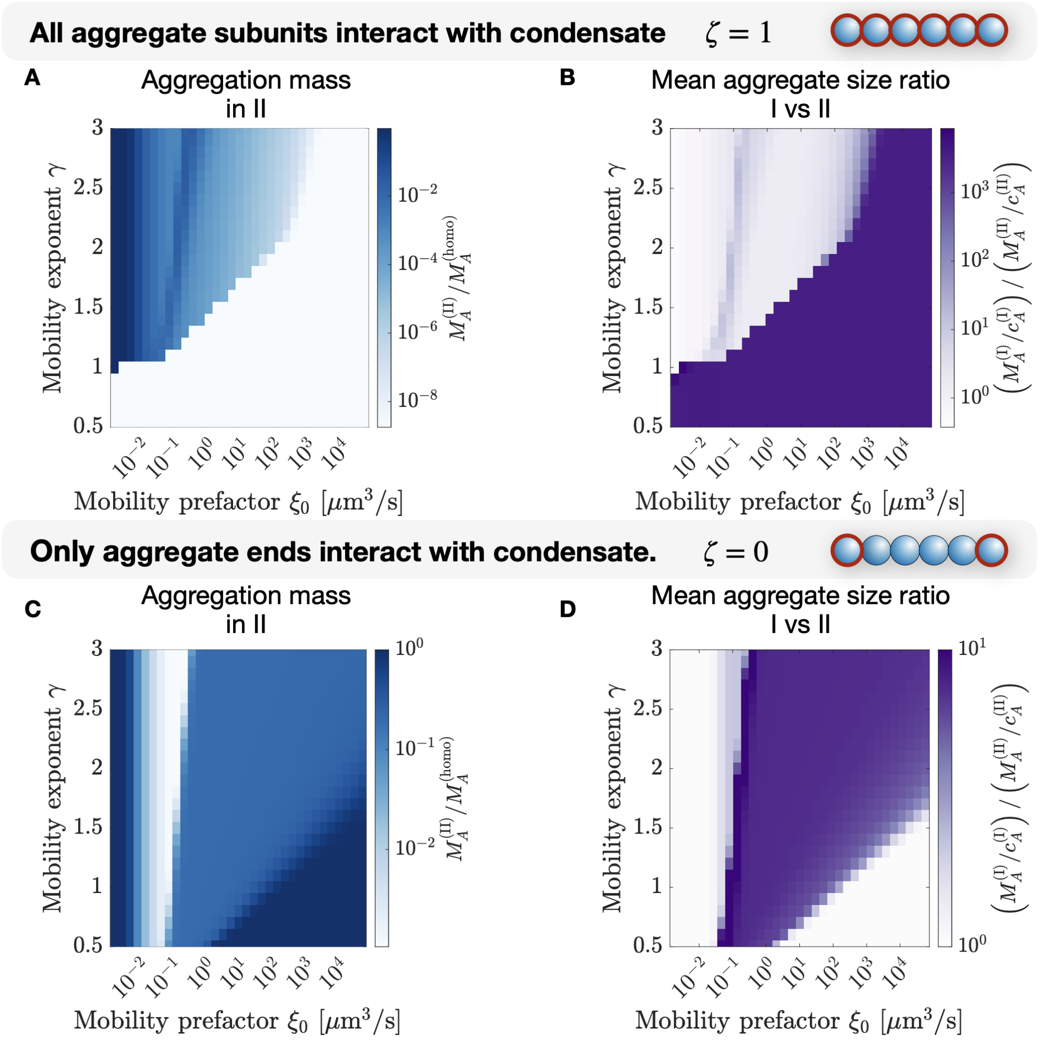

Figure S1: **Diagrams of how droplet rheology affects aggregate mass outside of the droplet and the size ratio of linear aggregates** For the two cases when all aggregate subunits interact with the condensate, ($\zeta = 1$, the partitioning coefficient is given by $P_i = \exp 2i$) or when only aggregate ends interact with the condensate ($\zeta = 0$, the partitioning coefficient $P_i = \exp 2$), we solve the master equation (see Eq. (1a) and Eq. (1b)) and here show aggregate mass concentration in compartment II after the time it took to assemble 99% monomers for (A) $\zeta = 1$ and (C) $\zeta = 0$. The mass concentration is normalised by mass concentration $M_{\mathrm{A}}^{(\mathrm{homo})}$ of the homogeneous case with only a single compartment with the same initial total monomer concentration. In (B,D), we provide the aggregate size ratio, where the size in compartment $\alpha = I, II$ results from $M_{\mathrm{A}}^{(\alpha)}/c_{\mathrm{A}}^{(\alpha)}$. For the solution of the master equation, we chose a total initial monomer concentration of $c_1^{(\mathrm{tot})}(t = 0) = 4\ \mu$M (see Eq. 13) and the volumes are $V^{(\mathrm{I})} = 10.1\ \mu\mathrm{m}^3$ and $V^{(\mathrm{II})} = 1000\ \mu\mathrm{m}^3$.

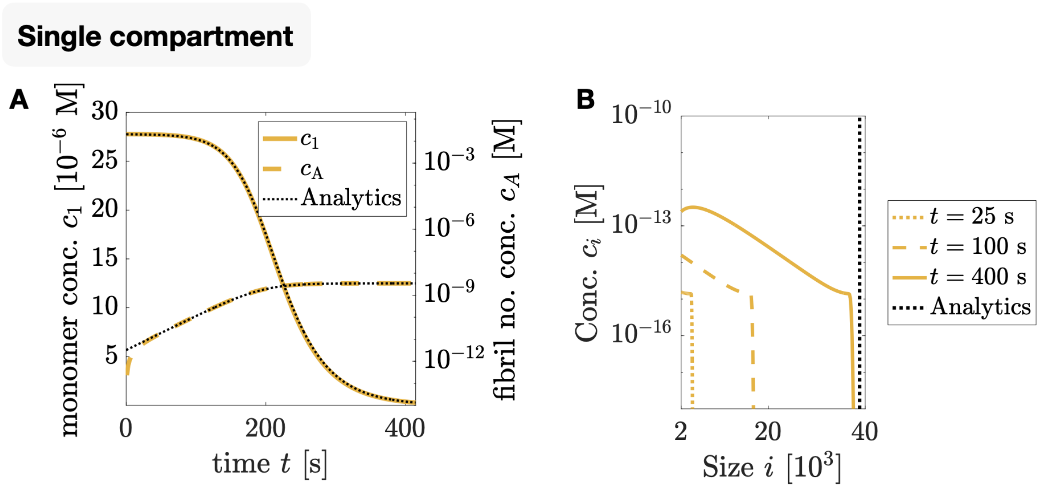

Figure S2: **Linear aggregate concentrations for aggregation in a homogeneous system** (A) Monomer concentration c_1 (solid line) and aggregate number concentration c_A (dashed line, see Eq. (2)) from the numerical solution of the master equation (Eq. (1a) and Eq. (1b)). Here, initially only monomers exist with a concentration $c_1(t = 0) = 27.78 \times 10^{-6}$ M to mimick a higher concentration within a droplet. The numerical solution is in excellent agreement with analytical predictions , see Eq. (S8) and Eq. (S9) (black dotted line). While the monomer concentration is decaying with time due to aggregation, the number of aggregates is increasing, until it saturates since almost all monomers are gone and primary nucleation no longer creates new fibrils. (B) Concentration profiles as a function of time. The profile possesses a moving front where the concentration decreases sharply. The black dotted line is the analytical prediction of the front position at the equilibrium, derived in Eq. 42.

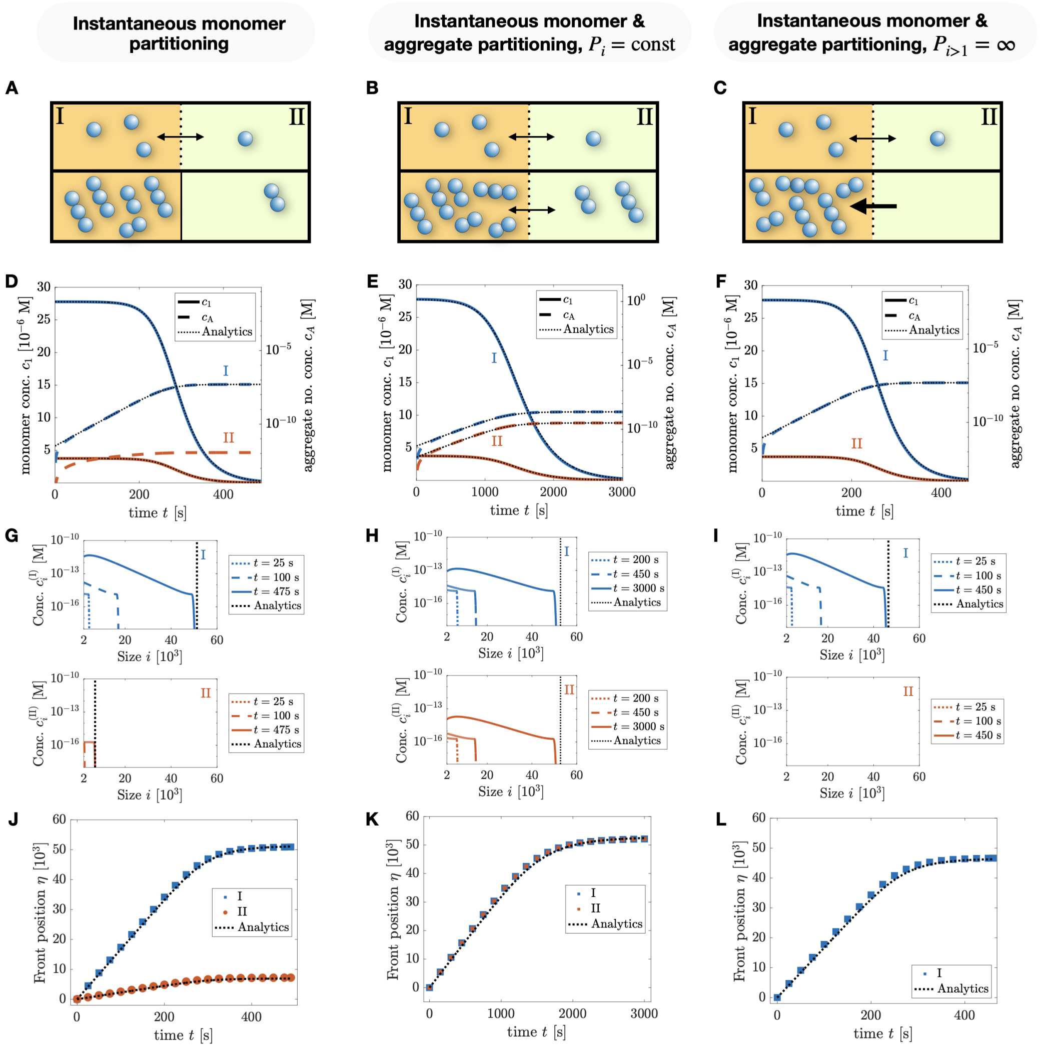

Figure S3: **Comparison of numerical and analytical solution for three different limits of instantaneous monomer and aggregate exchange** The three limits we are considering are: (A) Exclusive monomer exchange, (B) monomer and aggregate exchange with constant partitioning coefficient $P_i = \exp(2)$ and (C) monomer and aggregate exchange with monomer partitioning coefficient $P_1 = \exp(2)$ and aggregate partitioning coefficient $P_{i>1} = \infty$. In (D-F) we show the numerical solutions of the master equation (Eq. (1a) and Eq. (1b)) for the monomer concentration c_1 and the aggregate number concentration c_A for each compartment. The solutions are in excellent agreement with the analytical predictions, provided in Section S1.2.2 and Section S1.2.3.

Figure S3: (G-I)Concentration profiles as a function of time for the three cases in both compartments I (inside the droplet) and II (outside the droplet). The profiles possess a front where the concentrations decay abruptly. This front emerges from the fact that the aggregate assemble for a finite time and larger aggregates did not yet have time to form. The black dotted line corresponds to the position of the front in equilibrium, as predicted analytically. (J-L) Moving front position as a function of time from the numerical solution. How the front position is derived from the concentration profile is explained in Appendix A. he black dotted line corresponds to the analytical predictions. The total initial monomer concentration is $c_1^{(\mathrm{tot})}(t = 0) = 4\ \mu\mathrm{M}$ (see Eq. (14)) and the volumes are $V^{(\mathrm{I})} = 10.1\ \mu\mathrm{m}^3$ and $V^{(\mathrm{II})} = 1000\ \mu\mathrm{m}^3$.

S1 Analytical solutions for instantaneous exchange of monomers and linear aggregates

Before we derive the analytical solution of the linear aggregation of monomers and aggregates instantaneously exchanged between two phases, we first give the solutions for the single phase case, as previously derived in [1]. Then, we solve the analytical cases of exclusive and instantaneous monomer exchange, on time-scales much faster than the aggregation time-scale [2]. Finally, we solve the case with both, instantaneous monomer and aggregate exchange, and investigate the limit of infinite aggregate partitioning coefficient P_{f}.

S1.1 Single phase

Constitutive equations In a homogeneous system, with only a single phase, the aggregation dynamics is described by the equations

$$\begin{aligned} \frac{\mathrm{d}c_1(t)}{\mathrm{d}t} &= -2k_+c_1(t)c_{\mathrm{a}}(t) \\ &\quad -k_1n_1c_1(t)^{n_1} - k_2n_2c_1(t)^{n_2}M_{\mathrm{a}}(t) && \text{(S1a)} \\ \frac{\mathrm{d}c_{\mathrm{a}}(t)}{\mathrm{d}t} &= k_1c_1(t)^{n_1} + k_2c_1(t)^{n_2}M_{\mathrm{a}}(t) && \text{(S1b)} \end{aligned}$$

for the monomer concentration $c_1(t)$, the aggregate number concentration $c_{\mathrm{a}}(t)$ and the aggregate mass concentration $M_{\mathrm{a}}(t) = M_{\mathrm{tot}} - c_1(t)$. For typical filamentous systems, growth by elongation is much faster than primary and secondary nucleation [1]. In this regime, Eq. (S1a) and (S1b) can be simplified to

$$\begin{aligned} \frac{\mathrm{d}c_1(t)}{\mathrm{d}t} &= -2k_+c_1(t)c_{\mathrm{a}}(t) \ , && \text{(S2a)} \\ \frac{\mathrm{d}c_{\mathrm{a}}(t)}{\mathrm{d}t} &= k_1c_1(t)^{n_1} + k_2c_1(t)^{n_2}M_{\mathrm{a}}(t) && \text{(S2b)} \end{aligned}$$

where we have neglected nucleation terms in the equation for $\mathrm{d}c_1/\mathrm{d}t$. The initial conditions for Eq. (S2a) and (S2b) are $c_1(t=0) = M_{\mathrm{tot}}$ and $c_{\mathrm{a}}(t = 0) = 0$.

Analytical solutions for the monomer and aggregate number concentrations Equations (S2) has been solved analytically in [1] by using an

analogy to classical mechanics, rewriting the system of differential equations as

$$\frac{\mathrm{d}q}{\mathrm{d}t} = \frac{\partial \mathcal{H}}{\partial p}, \tag{S3a}$$

$$\frac{\mathrm{d}p}{\mathrm{d}t} = -\frac{\partial \mathcal{H}}{\partial q}, \tag{S3b}$$

with

$$p(t) = 2k_+ c_\mathrm{a}(t), \tag{S4a}$$

$$q(t) = \ln\left(\frac{M_\mathrm{tot}}{c_1(t)}\right), \tag{S4b}$$

$$\mathcal{H}(p,q) = \frac{p^2}{2} + V(q), \tag{S4c}$$

and the 'potential energy'

$$V(q) = \lambda^2 \frac{\exp(-n_1 q)}{n_1} + \kappa^2 \frac{\exp(-n_2 q)\left[(n_2+1) - n_2 \exp(-q)\right]}{n_2(n_2+1)}. \tag{S5}$$

Here, we introduce the parameters

$$\lambda = \sqrt{2k_+ k_1 M_\mathrm{tot}^{n_1}}, \tag{S6a}$$

$$\kappa = \sqrt{2k_+ k_2 M_\mathrm{tot}^{n_2+1}}, \tag{S6b}$$

$$\theta = \sqrt{\frac{2}{n_2\,(n_2+1)}}, \tag{S6c}$$

$$\omega = \frac{\lambda^2}{2\kappa^2\theta}, \tag{S6d}$$

assuming that at time $t = 0$, only monomers exist with the concentration $c_1(0) = M_\mathrm{tot}$. Using the mass conservation

$$M_\mathrm{a}(t) = c_1(0) - c_1(t), \tag{S7}$$

and solving the Euler-Lagrange equation $\frac{\mathrm{d}^2 q}{\mathrm{d}t^2} = -\partial_q V$, we can finally derive an approximate solution for the monomer concentration, given by (see Fig. S2*A*)

$$c_1(t) = M_\mathrm{tot}\left[1 + \omega \exp(\kappa t)\right]^{-\theta}. \tag{S8}$$

The aggregate number concentration $c_\mathrm{a}(t)$ results from Eq. (S2) and Eq. (S8) and is given by (see Fig. S2 *A*)

$$\begin{aligned} c_\mathrm{a}(t) &= -\frac{1}{2k_+c_1(t)}\frac{\mathrm{d}c_1}{\mathrm{d}t} \\ &= \frac{\theta\kappa}{2k_+}\frac{\omega\exp(\kappa t)}{1+\omega\exp(\kappa t)}, \end{aligned} \tag{S9}$$

which for $t \to \infty$ converges towards

$$\lim_{t\to\infty} c_\mathrm{a}(t) = \frac{\theta\kappa}{2k_+} \propto M_\mathrm{tot}^{\frac{n_2+1}{2}}. \tag{S10}$$

From Eq. (S8) we find that the characteristic rate of aggregation is given by

$$\kappa = \sqrt{2k_+k_2M_\mathrm{tot}^{n_2+1}} \propto M_\mathrm{tot}^{\frac{n_2+1}{2}}. \tag{S11}$$

Aggregation time Using Eq. (S8) and assuming that initially there are only monomers, after the time

$$T_\mathrm{p}^{(\mathrm{homo})} = \frac{1}{\kappa}\ln\left(\frac{(1-p)^{-\frac{1}{\theta}}-1}{\omega}\right) \tag{S12}$$

a fraction p of monomers have assembled. For example, if 99% of monomers have been used up, we set $p = 0.99$.

Moving front position We now introduce the variable

$$\eta(t) = \int_0^t \mathrm{d}t'\ 2k_+c_1(t') \tag{S13}$$

which corresponds to the average number of monomers used up by the growth of one aggregate at time t and hence is a measure of the largest linear aggregate size observed in the system. It is also a measure of the size for which the aggregate concentration profile is sharply declining, called the front (see Fig. S2 *B*). η can be calculated using Eq. (S9). The resulting expression

is in general complicated, involving hypergeometric functions. We can analytically solve it for $\theta = 0.5$, corresponding to $n_2 \approx 2.37$. Then, the front position η is given by

$$\begin{aligned}\eta(t) &= \frac{4k_+ M_{\text{tot}}}{\kappa}\left[\tanh^{-1}\left(\sqrt{1+\omega}\right)\right. \\ &\quad \left. -\tanh^{-1}\left(\sqrt{1+\omega\exp(\kappa t)}\right)\right], \qquad \text{(S14)}\\ \eta(\infty) &= \frac{4k_+ M_{\text{tot}}}{\kappa}\sinh^{-1}\left(\sqrt{\frac{1}{\omega}}\right). \qquad \text{(S15)}\end{aligned}$$

S1.2 Partitioning

We next consider the case where linear aggregates undergo aggregation within the phases I and II, but are also exchanged instantaneously between the phases by a total flux (see Eq. (11)). This corresponds to very high mobilities ξ_0 for monomers and ξ_f for aggregates.

Constitutive equations The dynamic equations of the monomer and aggregate number and mass concentrations, adapted from [2], are given by

$$\begin{aligned}\frac{\mathrm{d}c_1^{(\text{I})}(t)}{\mathrm{d}t} &= -2k_+c_1^{(\text{I})}(t)c_\text{a}^{(\text{I})}(t) - \xi_0\left[c_1^{(\text{I})}(t) - Pc_1^{(\text{II})}(t)\right]\frac{1}{V^{(\text{I})}}, \qquad \text{(S16a)}\\ \frac{\mathrm{d}c_1^{(\text{II})}(t)}{\mathrm{d}t} &= -2k_+c_1^{(\text{II})}(t)c_\text{a}^{(\text{II})}(t) + \xi_0\left[c_1^{(\text{I})}(t) - Pc_1^{(\text{II})}(t)\right]\frac{1}{V^{(\text{II})}}, \qquad \text{(S16b)}\\ \frac{\mathrm{d}M_\text{a}^{(\text{I})}(t)}{\mathrm{d}t} &= 2k_+c_1^{(\text{I})}(t)c_\text{a}^{(\text{I})}(t) - \xi_\text{f}\left[M_\text{a}^{(\text{I})}(t) - P_\text{f}M_\text{a}^{(\text{II})}(t)\right]\frac{1}{V^{(\text{I})}}, \qquad \text{(S16c)}\\ \frac{\mathrm{d}M_\text{a}^{(\text{II})}(t)}{\mathrm{d}t} &= 2k_+c_1^{(\text{II})}(t)c_\text{a}^{(\text{II})}(t) + \xi_\text{f}\left[M_\text{a}^{(\text{I})}(t) - P_\text{f}M_\text{a}^{(\text{II})}(t)\right]\frac{1}{V^{(\text{II})}}, \qquad \text{(S16d)}\\ \frac{\mathrm{d}c_\text{a}^{(\text{I})}(t)}{\mathrm{d}t} &= k_1 c_1^{(\text{I})}(t)^{n_1} + k_2 c_1^{(\text{I})}(t)^{n_2} M_\text{a}^{(\text{I})}(t)\\ &\quad -\xi_\text{f}\left[c_\text{a}^{(\text{I})}(t) - P_\text{f}c_\text{a}^{(\text{II})}(t)\right]\frac{1}{V^{(\text{I})}}, \qquad \text{(S16e)}\\ \frac{\mathrm{d}c_\text{a}^{(\text{II})}(t)}{\mathrm{d}t} &= k_1 c_1^{(\text{II})}(t)^{n_1} + k_2 c_1^{(\text{II})}(t)^{n_2} M_\text{a}^{(\text{II})}(t)\\ &\quad +\xi_\text{f}\left[c_\text{a}^{(\text{I})}(t) - P_\text{f}c_\text{a}^{(\text{II})}(t)\right]\frac{1}{V^{(\text{II})}} \qquad \text{(S16f)}\end{aligned}$$

where we introduced the aggregate partitioning coefficient P_{f}, mobility ξ_{f}, the monomer concentration $c_1^{(\alpha)}$, the aggregate number concentration $c_{\mathrm{a}}^{(\alpha)}$ and the aggregate mass concentration $M_{\mathrm{a}}^{(\alpha)}$, for $\alpha = \mathrm{I}, \mathrm{II}$. Here, we linearized the fluxes (see Eq. (11)). The sole task of the fluxes in the constitutive equations is to enforce the ratios of the number and mass concentrations ($c_1^{(\alpha)}$, $c_{\mathrm{a}}^{(\alpha)}$ and $M_{\mathrm{a}}^{(\alpha)}$) are equal to the partitioning coefficients P and P_{f}. Thus, the exact form of the fluxes has no impact on the analytical solutions.

Initial condition and mass conservation Here, and in the following, we will assume that initially only monomers exist with

$$c_1^{(\mathrm{I})}(0)V^{(\mathrm{I})} + c_1^{(\mathrm{II})}(0)V^{(\mathrm{II})} = M_{\mathrm{tot}}\left(V^{(\mathrm{I})} + V^{(\mathrm{II})}\right), \tag{S17}$$

where we introduce the total mass concentration M_{tot}. Together with the assumption that $c_1^{(\mathrm{I})}(0) = Pc_1^{(\mathrm{II})}(0)$ we get

$$c_1^{(\mathrm{I})}(0) = M_{\mathrm{tot}}\frac{V^{(\mathrm{I})} + V^{(\mathrm{II})}}{V^{(\mathrm{I})} + \frac{1}{P}V^{(\mathrm{II})}}, \tag{S18a}$$

$$c_1^{(\mathrm{II})}(0) = \frac{1}{P}M_{\mathrm{tot}}\frac{V^{(\mathrm{I})} + V^{(\mathrm{II})}}{V^{(\mathrm{I})} + \frac{1}{P}V^{(\mathrm{II})}}. \tag{S18b}$$

Mass conservation leads to

$$M_{\mathrm{a}}^{(\mathrm{I})}(t) = \frac{c_1^{(\mathrm{I})}(0)V^{(\mathrm{I})} + c_1^{(\mathrm{II})}(0)V^{(\mathrm{II})} - c_1^{(\mathrm{I})}(t)V^{(\mathrm{I})} - \left(c_1^{(\mathrm{II})}(t) + M_{\mathrm{a}}^{(\mathrm{II})}(t)\right)V^{(\mathrm{II})}}{V^{(\mathrm{I})}}, \tag{S19}$$

with the phase volumes $V^{(\mathrm{I})}$ and $V^{(\mathrm{II})}$.

We now provide analytical solutions to the constitutive equations in three limits of practical importance.

S1.2.1 No transport between phases I and II

If partitioning between the phases is prohibited, corresponding to $\xi_0 = 0$ and $\xi_{\mathrm{f}} = 0$, in each phase the aggregation is equivalent to the aggregation dynamics in the homogeneous case (see Section S1.1) with the initial monomer concentrations $c_1^{(\mathrm{I})}(0)$ in phase I and $c_1^{(\mathrm{II})}(0)$ in phase II.

S1.2.2 Rapid transport of monomers between two phases

This case has been previously investigated in [2] and is sketched in Fig. S3 *A*.

Assumptions Here, we assume that only monomers are transported on such a short time-scale, that at any time the condition

$$c_1^{(\mathrm{I})}(t) = P c_1^{(\mathrm{II})}(t) \tag{S20}$$

is fulfilled. There is no exchange of aggregates between the two phases. Additionally, we choose the initial conditions and P such that within phase II the monomer concentration is continuously smaller than in phase I, so that the aggregate mass and number concentrations in II are negligible, $M_\mathrm{a}^{(\mathrm{II})} \approx 0$ and $c_\mathrm{a}^{(\mathrm{II})} \approx 0$.

Analytical solutions for the monomer and aggregate number concentrations The total monomer concentration in the system follows from

$$c_1(t) = \frac{V^{(\mathrm{I})}}{V^{(\mathrm{I})} + V^{(\mathrm{II})}} c_1^{(\mathrm{I})}(t) + \frac{V^{(\mathrm{II})}}{V^{(\mathrm{I})} + V^{(\mathrm{II})}} c_1^{(\mathrm{II})}(t), \tag{S21}$$

$$\frac{\mathrm{d}c_1(t)}{\mathrm{d}t} = \frac{V^{(\mathrm{I})}}{V^{(\mathrm{I})} + V^{(\mathrm{II})}} \frac{\mathrm{d}c_1^{(\mathrm{I})}(t)}{\mathrm{d}t} + \frac{V^{(\mathrm{II})}}{V^{(\mathrm{I})} + V^{(\mathrm{II})}} \frac{\mathrm{d}c_1^{(\mathrm{II})}(t)}{\mathrm{d}t}. \tag{S22}$$

By using the enforced ratio of monomers in phase I and II, see Eq. (S20), we find

$$\frac{\mathrm{d}c_1(t)}{\mathrm{d}t} = \frac{\mathrm{d}c_1^{(\mathrm{I})}(t)}{\mathrm{d}t} \frac{V^{(\mathrm{I})} + \frac{1}{P} V^{(\mathrm{II})}}{V^{(\mathrm{I})} + V^{(\mathrm{II})}}. \tag{S23}$$

Additionally, combining Eq. (S22) with the constitutive equations, Eq. (S16a) and Eq. (S16b), and assuming that $c_\mathrm{a}^{(\mathrm{II})}(t) \approx 0$, we can also write

$$\frac{\mathrm{d}c_1(t)}{\mathrm{d}t} = -2k_+ c_1^{(\mathrm{I})} c_\mathrm{a}^{(\mathrm{I})} \frac{V^{(\mathrm{I})}}{V^{(\mathrm{I})} + V^{(\mathrm{II})}}. \tag{S24}$$

Combining Eq. (S23) and Eq. (S24) finally leads to

$$\frac{\mathrm{d}c_1^{(\mathrm{I})}(t)}{\mathrm{d}t} = -2k_+ c_1^{(\mathrm{I})} c_\mathrm{a}^{(\mathrm{I})} \frac{1}{1 + \frac{1}{P} \frac{V^{(\mathrm{II})}}{V^{(\mathrm{I})}}}. \tag{S25}$$

The aggregate number concentration in phase I follows from Eq. (S16e), given by

$$\frac{\mathrm{d}c_\mathrm{a}^{(\mathrm{I})}(t)}{\mathrm{d}t} = k_1 {c_1^{(\mathrm{I})}}^{n_1} + k_2 {c_1^{(\mathrm{I})}}^{n_2} \left(1 + \frac{1}{P} \frac{V^{(\mathrm{II})}}{V^{(\mathrm{I})}}\right) \left(c_1^{(\mathrm{I})}(0) - c_1^{(\mathrm{I})}(t)\right) \tag{S26}$$

where we have used $M_{\mathrm{a}}^{(\mathrm{II})}(t) \approx 0$ and the mass conservation (see Eq. (S19))

$$
\begin{aligned}
M_{\mathrm{a}}^{(\mathrm{I})}(t) &= \frac{c_1^{(\mathrm{I})}(0)V^{(\mathrm{I})} + c_1^{(\mathrm{II})}(0)V^{(\mathrm{II})} - c_1^{(\mathrm{I})}(t)V^{(\mathrm{I})} - c_1^{(\mathrm{II})}(t)V^{(\mathrm{II})}}{V^{(\mathrm{I})}} \\
&= \left(1 + \frac{1}{P}\frac{V^{(\mathrm{II})}}{V^{(\mathrm{I})}}\right)\left(c_1^{(\mathrm{I})}(0) - c_1^{(\mathrm{I})}(t)\right). \qquad \text{(S27)}
\end{aligned}
$$

The equations (S25) and (S26) have the same form as the homogeneous case introduced in Section S1.1 with the effective rate constants

$$
\begin{aligned}
\tilde{k}_1 &= k_1, && \text{(S28a)} \\
\tilde{k}_2 &= k_2\left(1 + \frac{1}{P}\frac{V^{(\mathrm{II})}}{V^{(\mathrm{I})}}\right), && \text{(S28b)} \\
\tilde{k}_+ &= k_+\frac{1}{1 + \frac{1}{P}\frac{V^{(\mathrm{II})}}{V^{(\mathrm{I})}}}. && \text{(S28c)}
\end{aligned}
$$

The monomer concentrations are then given by

$$
\begin{aligned}
c_1^{(\mathrm{I})}(t) &= c_1^{(\mathrm{I})}(0)\left[1 + \omega\exp(\kappa t)\right]^{-\theta}, && \text{(S29a)} \\
c_1^{(\mathrm{II})}(t) &= \frac{1}{P}c_1^{(\mathrm{I})}(0)\left[1 + \omega\exp(\kappa t)\right]^{-\theta} && \text{(S29b)}
\end{aligned}
$$

with the parameters

$$
\begin{aligned}
\lambda &= \sqrt{2\tilde{k}_+\tilde{k}_1 c_1^{(\mathrm{I})}(0)^{n_1}}, && \text{(S30a)} \\
\kappa &= \sqrt{2\tilde{k}_+\tilde{k}_2 c_1^{(\mathrm{I})}(0)^{n_2+1}}, && \text{(S30b)} \\
\theta &= \sqrt{\frac{2}{n_2\left(n_2+1\right)}}, && \text{(S30c)} \\
\omega &= \frac{\lambda^2}{2\kappa^2\theta}. && \text{(S30d)}
\end{aligned}
$$

The aggregate number concentration in phase I then results from Eq. (S16a) and Eq. (S29a) and is given by

$$
c_{\mathrm{a}}^{(\mathrm{I})}(t) = \frac{1}{\omega + \exp(-\kappa t)}\frac{\theta\omega\kappa}{2\tilde{k}_+} \qquad \text{(S31)}
$$

and

$$\lim_{t\to\infty} c_{\mathrm{a}}^{(\mathrm{I})}(t) = \frac{\theta\kappa}{2\tilde{k}_+}. \tag{S32}$$

The total aggregate number concentration $c_{\mathrm{a}}(t)$ in the system is given by

$$\begin{aligned} c_{\mathrm{a}}(t) &= \frac{V^{(\mathrm{I})}c_{\mathrm{a}}^{(\mathrm{I})}(t) + V^{(\mathrm{II})}c_{\mathrm{a}}^{(\mathrm{II})}(t)}{V^{(\mathrm{I})} + V^{(\mathrm{II})}} \\ &\approx \frac{V^{(\mathrm{I})}}{V^{(\mathrm{I})} + V^{(\mathrm{II})}} c_{\mathrm{a}}^{(\mathrm{I})}(t) \\ &= \frac{V^{(\mathrm{I})}}{V^{(\mathrm{I})} + V^{(\mathrm{II})}} \frac{1}{\omega + \exp(-\kappa t)} \frac{\theta\omega\kappa}{2\tilde{k}_+}, \end{aligned} \tag{S33}$$

and

$$\lim_{t\to\infty} c_{\mathrm{a}}(t) = \frac{V^{(\mathrm{I})}}{V^{(\mathrm{I})} + V^{(\mathrm{II})}} \frac{\theta\kappa}{2\tilde{k}_+}. \tag{S34}$$

The analytical predictions are compared with numerical solutions of the master equation (see Eq. (1a) and Eq. (1b)) in Figure S3 *D* and shows excellent agreement.

Aggregation time Using Eq. (S21), Eq. (S29a) and Eq. (S29b), we derive the time

$$T_{\mathrm{p}} = \frac{1}{\kappa} \ln\left(\frac{(1+\omega)(1-p)^{-\frac{1}{\theta}} - 1}{\omega} \right) \tag{S35}$$

to assemble the fraction p of monomers into linear aggregates.

Moving front positions The front position of the moving front in phases I and II are derived analogously to the case of a single phase (see section S1.1) with

$$\eta^{(\mathrm{I})}(t) = \int_0^t \mathrm{d}t'\, 2k_+ c_1^{(\mathrm{I})}(t'), \tag{S36a}$$

$$\eta^{(\mathrm{II})}(t) = \int_0^t \mathrm{d}t'\, 2k_+ c_1^{(\mathrm{II})}(t'), \tag{S36b}$$

and the solution for $\theta = 0.5$ is given by

$$\eta^{(\mathrm{I})}(t) = \frac{4\tilde{k}_+ c_1^{(\mathrm{I})}(0)}{\kappa}\left[\tanh^{-1}\left(\sqrt{1+\omega}\right) - \tanh^{-1}\left(\sqrt{1+\omega\exp(\kappa t)}\right)\right], \quad \text{(S37a)}$$

$$\eta^{(\mathrm{II})}(t) = \frac{4\tilde{k}_+ c_1^{(\mathrm{I})}(0)}{P\kappa}\left[\tanh^{-1}\left(\sqrt{1+\omega}\right) - \tanh^{-1}\left(\sqrt{1+\omega\exp(\kappa t)}\right)\right], \quad \text{(S37b)}$$

$$\eta^{(\mathrm{I})}(\infty) = \frac{4\tilde{k}_+ c_1^{(\mathrm{I})}(0)}{\kappa}\sinh^{-1}\left(\sqrt{\frac{1}{\omega}}\right), \quad \text{(S37c)}$$

$$\eta^{(\mathrm{II})}(\infty) = \frac{4\tilde{k}_+ c_1^{(\mathrm{I})}(0)}{P\kappa}\sinh^{-1}\left(\sqrt{\frac{1}{\omega}}\right). \quad \text{(S37d)}$$

In Figure S3 *G* we solved the master equation numerically for infinitely high mobilities and show the aggregate concentration profiles as a function of time. The front is clearly visible for both phases and the temporal evolution of its position is in agreement with the analytical predictions, as shown in Fig. S3 *J*.

S1.2.3 Rapid transport of monomers and linear aggregates between phases I and II

Assumptions We now assume that not only the monomers are rapidly exchanged between the phase with the partitioning coefficient P, but also the linear aggregates with P_f, where P_f is independent of the aggregate size (see Fig. S3 *B*). Then,

$$c_1^{(\mathrm{I})}(t) = P c_1^{(\mathrm{II})}(t), \quad \text{(S38a)}$$

$$c_\mathrm{A}^{(\mathrm{I})}(t) = P_\mathrm{f} c_\mathrm{A}^{(\mathrm{II})}(t), \quad \text{(S38b)}$$

$$M_\mathrm{A}^{(\mathrm{I})}(t) = P_\mathrm{f} M_\mathrm{A}^{(\mathrm{II})}(t). \quad \text{(S38c)}$$

Additionally we assume that initially, no aggregates are present in the system, corresponding to $M_\mathrm{a}^{(\alpha)}(t=0) = 0$ and $c_\mathrm{a}^{(\alpha)}(t=0) = 0$ for $\alpha = \mathrm{I}, \mathrm{II}$.

Analytical solutions for the monomer and aggregate number concentrations Analogously to the previous section, we consider the total monomer concentration c_1 (see Eq. (S21) and Eq. (S22)), leading to

$$\frac{\mathrm{d}c_1(t)}{\mathrm{d}t} = \frac{\mathrm{d}c_1^{(\mathrm{I})}(t)}{\mathrm{d}t} \frac{V^{(\mathrm{I})} + \frac{1}{P}V^{(\mathrm{II})}}{V^{(\mathrm{I})} + V^{(\mathrm{II})}}. \tag{S39}$$

Additionally, combining

$$\frac{\mathrm{d}c_1(t)}{\mathrm{d}t} = \frac{\mathrm{d}c_1^{(\mathrm{I})}(t)}{\mathrm{d}t} \frac{V^{(\mathrm{I})}}{V^{(\mathrm{I})} + V^{(\mathrm{II})}} + \frac{\mathrm{d}c_1^{(\mathrm{II})}(t)}{\mathrm{d}t} \frac{V^{(\mathrm{II})}}{V^{(\mathrm{I})} + V^{(\mathrm{II})}} \tag{S40}$$

with Eq. (S16a) and Eq. (S16b) results in

$$\frac{\mathrm{d}c_1(t)}{\mathrm{d}t} = -2k_+ c_1^{(\mathrm{I})} c_\mathrm{a}^{(\mathrm{I})} \frac{V^{(\mathrm{I})} + \frac{1}{P}\frac{1}{P_\mathrm{f}}V^{(\mathrm{II})}}{V^{(\mathrm{I})} + V^{(\mathrm{II})}}. \tag{S41}$$

By comparing Eq. (S39) and Eq. (S41), we derive

$$\frac{\mathrm{d}c_1^{(\mathrm{I})}(t)}{\mathrm{d}t} = -2k_+ c_1^{(\mathrm{I})} c_\mathrm{a}^{(\mathrm{I})} \frac{V^{(\mathrm{I})} + \frac{1}{P}\frac{1}{P_\mathrm{f}}V^{(\mathrm{II})}}{V^{(\mathrm{I})} + \frac{1}{P}V^{(\mathrm{II})}}. \tag{S42}$$

By considering the total aggregate number concentration

$$c_\mathrm{a}(t) = \frac{V^{(\mathrm{I})}}{V^{(\mathrm{I})} + V^{(\mathrm{II})}} c_\mathrm{a}^{(\mathrm{I})}(t) + \frac{V^{(\mathrm{II})}}{V^{(\mathrm{I})} + V^{(\mathrm{II})}} c_\mathrm{a}^{(\mathrm{II})}(t), \tag{S43}$$

$$\frac{\mathrm{d}c_\mathrm{a}(t)}{\mathrm{d}t} = \frac{V^{(\mathrm{I})}}{V^{(\mathrm{I})} + V^{(\mathrm{II})}} \frac{\mathrm{d}c_\mathrm{a}^{(\mathrm{I})}(t)}{\mathrm{d}t} + \frac{V^{(\mathrm{II})}}{V^{(\mathrm{I})} + V^{(\mathrm{II})}} \frac{\mathrm{d}c_\mathrm{a}^{(\mathrm{II})}(t)}{\mathrm{d}t}, \tag{S44}$$

which, by using Eq (S38b), leads to

$$\frac{\mathrm{d}c_\mathrm{a}(t)}{\mathrm{d}t} = \frac{\mathrm{d}c_\mathrm{a}^{(\mathrm{I})}(t)}{\mathrm{d}t} \frac{V^{(\mathrm{I})} + \frac{1}{P_\mathrm{f}}V^{(\mathrm{II})}}{V^{(\mathrm{I})} + V^{(\mathrm{II})}}. \tag{S45}$$

Combining Eq. (S44), Eq. (S16e) and Eq. (S16f), we get

$$\frac{\mathrm{d}c_\mathrm{a}(t)}{\mathrm{d}t} = k_1 {c_1^{(\mathrm{I})}}^{n_1} \frac{V^{(\mathrm{I})} + \frac{1}{P^{n_1}}V^{(\mathrm{II})}}{V^{(\mathrm{I})} + V^{(\mathrm{II})}} + k_2 {c_1^{(\mathrm{I})}}^{n_2} M_\mathrm{a}^{(\mathrm{I})} \frac{V^{(\mathrm{I})} + \frac{1}{P^{n_2}}\frac{1}{P_\mathrm{f}}V^{(\mathrm{II})}}{V^{(\mathrm{I})} + V^{(\mathrm{II})}}. \tag{S46}$$

Since the mass is conserved, we get

$$V^{(\mathrm{I})}\left(c_1^{(\mathrm{I})}(t) + M_\mathrm{a}^{(\mathrm{I})}(t)\right) + V^{(\mathrm{II})}\left(c_1^{(\mathrm{II})}(t) + M_\mathrm{a}^{(\mathrm{II})}(t)\right) = \mathrm{const.} \tag{S47}$$

and if $M_{\mathrm{a}}^{(\mathrm{I})}(0)=0$ and $M_{\mathrm{a}}^{(\mathrm{II})}(0)=0$, this results in

$$M_{\mathrm{a}}^{(\mathrm{I})}(t)=\frac{V^{(\mathrm{I})}+\frac{1}{P}V^{(\mathrm{II})}}{V^{(\mathrm{I})}+\frac{1}{P_{\mathrm{f}}}V^{(\mathrm{II})}}\left(c_1^{(\mathrm{I})}(0)-c_1^{(\mathrm{I})}(t)\right). \tag{S48}$$

From Eq. (S48), Eq. (S45) and Eq. (S46) we then get

$$\begin{aligned}\frac{\mathrm{d}c_{\mathrm{a}}^{(\mathrm{I})}(t)}{\mathrm{d}t} &= k_1 {c_1^{(\mathrm{I})}}^{n_1}\frac{V^{(\mathrm{I})}+\frac{1}{P^{n_1}}V^{(\mathrm{II})}}{V^{(\mathrm{I})}+\frac{1}{P_{\mathrm{f}}}V^{(\mathrm{II})}}+k_2 {c_1^{(\mathrm{I})}}^{n_1}\left[c_1^{(\mathrm{I})}(0)-c_1^{(\mathrm{I})}(t)\right]\\ &\times\frac{V^{(\mathrm{I})}+\frac{1}{P^{n_2}}\frac{1}{P_{\mathrm{f}}}V^{(\mathrm{II})}}{V^{(\mathrm{I})}+\frac{1}{P_{\mathrm{f}}}V^{(\mathrm{I})}}\frac{V^{(\mathrm{I})}+\frac{1}{P}V^{(\mathrm{II})}}{V^{(\mathrm{I})}+\frac{1}{P_{\mathrm{f}}}V^{(\mathrm{II})}}.\end{aligned} \tag{S49}$$

Again, we use the fact that the equations (S42) and (S49) have the same form as in the single phase case introduced in Section S1.1 and can define the effective rate constants

$$\tilde{k}_1 = k_1\frac{V^{(\mathrm{I})}+\frac{1}{P^{n_1}}V^{(\mathrm{II})}}{V^{(\mathrm{I})}+\frac{1}{P_{\mathrm{f}}}V^{(\mathrm{II})}}, \tag{S50a}$$

$$\tilde{k}_2 = k_2\frac{V^{(\mathrm{I})}+\frac{1}{P^{n_2}}\frac{1}{P_{\mathrm{f}}}V^{(\mathrm{II})}}{V^{(\mathrm{I})}+\frac{1}{P_{\mathrm{f}}}V^{(\mathrm{I})}}\frac{V^{(\mathrm{I})}+\frac{1}{P}V^{(\mathrm{II})}}{V^{(\mathrm{I})}+\frac{1}{P_{\mathrm{f}}}V^{(\mathrm{II})}}, \tag{S50b}$$

$$\tilde{k}_+ = k_+\frac{V^{(\mathrm{I})}+\frac{1}{P}\frac{1}{P_{\mathrm{f}}}V^{(\mathrm{II})}}{V^{(\mathrm{I})}+\frac{1}{P}V^{(\mathrm{II})}}. \tag{S50c}$$

The solution is given by

$$c_1^{(\mathrm{I})}(t) = c_1^{(\mathrm{I})}(0)\left[1+\omega\exp(\kappa t)\right]^{-\theta}, \tag{S51a}$$

$$c_1^{(\mathrm{II})}(t) = \frac{1}{P}c_1^{(\mathrm{I})}(0)\left[1+\omega\exp(\kappa t)\right]^{-\theta}. \tag{S51b}$$

with the parameters

$$\lambda = \sqrt{2\tilde{k}_+\tilde{k}_1\left(c_1^{(\mathrm{I})}(0)\right)^{n_1}}, \tag{S52a}$$

$$\kappa = \sqrt{2\tilde{k}_+\tilde{k}_2\left(c_1^{(\mathrm{I})}(0)\right)^{n_2+1}}, \tag{S52b}$$

$$\theta = \sqrt{\frac{2}{n_2\left(n_2+1\right)}}, \tag{S52c}$$

$$\omega = \frac{\lambda^2}{2\kappa^2\theta}. \tag{S52d}$$

The aggregate number concentration inside and outside of the condensate results from Eq. (S16a) and Eq. (S51a) and is then given by

$$c_{\mathrm{a}}^{(\mathrm{I})}(t) = \frac{1}{\omega + \exp(-\kappa t)} \frac{\theta\omega\kappa}{2\tilde{k}_{+}}, \tag{S53a}$$

$$c_{\mathrm{a}}^{(\mathrm{II})}(t) = \frac{1}{P_{\mathrm{f}}} \frac{1}{\omega + \exp(-\kappa t)} \frac{\theta\omega\kappa}{2\tilde{k}_{+}} \tag{S53b}$$

converging to

$$\lim_{t\to\infty} c_{\mathrm{a}}^{(\mathrm{I})}(t) = \frac{\theta\kappa}{2\tilde{k}_{+}} \tag{S54a}$$

$$\lim_{t\to\infty} c_{\mathrm{a}}^{(\mathrm{II})}(t), = \frac{1}{P_{\mathrm{f}}} \frac{\theta\kappa}{2\tilde{k}_{+}}. \tag{S54b}$$

The total aggregate number concentration in the system is then given by

$$c_{\mathrm{a}}(t) = \frac{V^{(\mathrm{I})} + \frac{1}{P_{\mathrm{f}}} V^{(\mathrm{II})}}{V^{(\mathrm{I})} + V^{(\mathrm{II})}} \frac{1}{\omega + \exp(-\kappa t)} \frac{\theta\omega\kappa}{2\tilde{k}_{+}} \tag{S55}$$

and

$$\lim_{t\to\infty} c_{\mathrm{a}}(t) = \frac{V^{(\mathrm{I})} + \frac{1}{P_{\mathrm{f}}} V^{(\mathrm{II})}}{V^{(\mathrm{I})} + V^{(\mathrm{II})}} \frac{\theta\kappa}{2\tilde{k}_{+}}. \tag{S56}$$

In Fig. S3 *E* we compare our analytical predictions for the monomer concentration and the aggregate number concentration with the numerical solution of the master equation for infinitely high mobilities and we find that both are in excellent agreement.

Aggregation time It takes the time

$$T_{\mathrm{p}} = \frac{1}{\kappa} \ln\left(\frac{(1+\omega)(1-p)^{-\frac{1}{\theta}} - 1}{\omega} \right) \tag{S57}$$

to assemble a fraction p of all monomers.

Moving front positions The front position of the moving front in phase I is derived analogously to the case of a single phase (see section S1.1) with

$$\eta^{(\mathrm{I})}(t) = \int_0^t \mathrm{d}t' \; 2\tilde{k}_{+} c_1^{(\mathrm{I})}(t'). \tag{S58}$$

Note that here we use $\tilde{k}_+$ instead of k_+ since the aggregate concentrations in both phases are coupled (see Eq. (S38b)). The solution for $\theta = 0.5$ is given by

$$\begin{aligned}\eta^{(\mathrm{I})}(t) &= \frac{4\tilde{k}_+ c_1^{(\mathrm{I})}(0)}{\kappa}\left[\tanh^{-1}\left(\sqrt{1+\omega}\right)\right.\\ &\qquad \left.-\tanh^{-1}\left(\sqrt{1+\omega\exp\left(\kappa t\right)}\right)\right], \qquad \text{(S59)}\\ \eta^{(\mathrm{I})}(\infty) &= \frac{4\tilde{k}_+ c_1^{(\mathrm{I})}(0)}{\kappa}\sinh^{-1}\left(\sqrt{\frac{1}{\omega}}\right). \qquad \text{(S60)}\end{aligned}$$

Since for $P > 1$ and $P_\mathrm{f} > 1$, in phase I we expect more material, the front position in this phase will also be larger. Since we allow for rapid material exchange between the inside and outside of the condensate, the front will be located at the same location in phase II as in phase I:

$$\eta^{(\mathrm{II})}(t) = \eta^{(\mathrm{I})}(t). \qquad \text{(S61)}$$

This result can be confirmed by numerically solving the master equation and investigating the aggregate concentration profile (see Fig. S3 *H* and Fig. S3 *K*) and indeed, the front positions are identical for both phases.

Behaviour in the limit $P_\mathrm{f} \to \infty$ For large aggregate partitioning coefficient $P_\mathrm{f} \to \infty$ (see Fig. S3 *C*), the effective rate constants are

$$\begin{aligned}\tilde{k}_1 &= k_1\left(1 + \frac{1}{P^{n_1}}\frac{V^{(\mathrm{II})}}{V^{(\mathrm{I})}}\right), \qquad \text{(S62a)}\\ \tilde{k}_2 &= k_2\left(1 + \frac{1}{P}\frac{V^{(\mathrm{II})}}{V^{(\mathrm{I})}}\right), \qquad \text{(S62b)}\\ \tilde{k}_+ &= k_+\frac{1}{1+\frac{1}{P}\frac{V^{(\mathrm{II})}}{V^{(\mathrm{I})}}}. \qquad \text{(S62c)}\end{aligned}$$

The rate constants $\tilde{k}_2$ and $\tilde{k}_+$ are identical to the case without aggregate exchange between the phases, see Eq. (S28b) and Eq. (S28c). Thus, the characteristic time of aggregation κ is identical for these two cases. With these rate constants, the monomer concentration and aggregate number concentration is in agreement with the analytical predictions for general P_f (see Fig. S3 *F*).

The front position results from

$$\eta^{(\mathrm{I})}(t) = \int_0^t \mathrm{d}t' \; 2k_+ c_1^{(\mathrm{I})}(t'). \tag{S63}$$

Note that for the calculation of κ and λ we use $\tilde{k}_+$, but in the front position integral we use k_+. This results from the fact that the only location where aggregates exist and thus, linear aggregate elongation takes place, is located in phase I and we can treat it as a single phase (see Appendix S1.1). For $\theta = 0.5$, the front is then given by

$$\begin{aligned} \eta^{(\mathrm{I})}(t) &= \frac{4k_+ c_1^{(\mathrm{I})}(0)}{\kappa}\Big[\tanh^{-1}\left(\sqrt{1+\omega}\right) \\ &\quad - \tanh^{-1}\left(\sqrt{1+\omega\exp\left(\kappa t\right)}\right)\Big], \qquad \text{(S64)} \\ \eta^{(\mathrm{I})}(\infty) &= \frac{4k_+ c_1^{(\mathrm{I})}(0)}{\kappa}\sinh^{-1}\left(\sqrt{\frac{1}{\omega}}\right). \qquad \text{(S65)} \end{aligned}$$

In phase II we assume that for $P_\mathrm{f} \to \infty$ no aggregates can be found and thus, no front exists. This can be confirmed with numerical solutions of the master equation (see Fig. S3 *I* and Fig. S3 *L*).

References

[1] T. C. Michaels, S. I. Cohen, M. Vendruscolo, C. M. Dobson, and T. P. Knowles, "Hamiltonian dynamics of protein filament formation," *Phys Rev Lett*, vol. 116, no. 3, p. 038101, 2016.

[2] C. Weber, T. Michaels, and L. Mahadevan, "Spatial control of irreversible protein aggregation," *eLife*, vol. 8, p. e42315, 2019.
